# Intracellular cartilage oligomeric matrix protein augments breast cancer resistance to chemotherapy

**DOI:** 10.1038/s41419-024-06872-7

**Published:** 2024-07-04

**Authors:** Veroniaina Hanitrarimalala, Izabela Bednarska, Takashi Murakami, Konstantinos S. Papadakos, Anna M. Blom

**Affiliations:** 1 Department of Translational Medicine, Lund University, Malmö, S-214 28 Sweden; 2 Department of Microbiology, Saitama Medical University, Saitama, 350-0495 Japan

**Keywords:** Breast cancer, Cancer microenvironment

## Abstract

Chemotherapy persists as the primary intervention for breast cancer, with chemoresistance posing the principal obstacle to successful treatment. Herein, we show that cartilage oligomeric matrix protein (COMP) expression leads to increased cancer cell survival and attenuated apoptosis under treatment with several chemotherapeutic drugs, anti-HER2 targeted treatment, and endocrine therapy in several breast cancer cell lines tested. The COMP-induced chemoresistance was independent of the breast cancer subtype. Extracellularly delivered recombinant COMP failed to rescue cells from apoptosis while endoplasmic reticulum (ER)-restricted COMP-KDEL conferred resistance to apoptosis, consistent with the localization of COMP in the ER, where it interacted with calpain. Calpain activation was reduced in COMP-expressing cells and maintained at a lower level of activation during treatment with epirubicin. Moreover, the downstream caspases of calpain, caspases -9, -7, and -3, exhibited significantly reduced activation in COMP-expressing cells under chemotherapy treatment. Chemotherapy, when combined with calpain activators, rendered the cells expressing COMP more chemosensitive. Also, the anti-apoptotic proteins phospho-Bcl2 and survivin were increased in COMP-expressing cells upon chemotherapy. Cells expressing a mutant COMP lacking thrombospondin repeats exhibited reduced chemoresistance compared to cells expressing full-length COMP. Evaluation of calcium levels in the ER, cytosol, and mitochondria revealed that COMP expression modulates intracellular calcium homeostasis. Furthermore, patients undergoing chemotherapy or endocrine therapy demonstrated significantly reduced overall survival time when tumors expressed high levels of COMP. This study identifies a novel role of COMP in chemoresistance and calpain inactivation in breast cancer, a discovery with potential implications for anti-cancer therapy.

## Introduction

Breast cancer is the leading cause of cancer-related mortality in women. Chemotherapy, radiotherapy, endocrine therapy, and HER2-targeted therapy are the most common treatment options. However, an increase of mortality is observed in breast cancer patients due to chemoresistance. Thus, understanding the mechanisms underlying chemoresistance is critical for overcoming this clinical challenge.

Cartilage oligomeric matrix protein (COMP), also known as thrombospondin-5 (TSP-5), is an extracellular matrix (ECM) protein found primarily in the cartilage, where it influences the structure of ECM during tissue development and remodeling. COMP is a pentameric glycoprotein, with each monomer consisting of an N-terminal polymerization region (NTR), four type-2 epidermal growth factor (EGF)-like domains, eight type-3 thrombospondin repeats (TSP) and a globular C-terminal region (CTR). We discovered that COMP, which is normally highly restricted, is expressed in breast cancer, prostate cancer, periampullary adenocarcinoma, and colorectal cancer [1–4], while other groups reported its expression in colon, thyroid, and urothelial carcinoma [5–7]. High levels of COMP were repeatedly linked to larger tumor size, metastasis, faster cancer recurrence, and shorter overall survival. High levels of COMP were also detected in the sera of patients with metastatic breast cancer [8, 9]. Mechanistically, COMP increases the invasiveness of breast cancer cells by increasing MMP9 expression, while having no effects on adhesion and migration in vitro [1]. COMP also promotes cancer cell stemness by increasing the interaction between Notch3 and its ligand Jagged1, resulting in increased activation of Jagged1-Notch3 signaling and cross-reactivity with other important cancer-related pathways, such as AKT and β-catenin [10].

Chemoresistance of cancer cells has complex mechanisms that have yet to be fully understood. Resistance to chemotherapeutic drugs may exist in cancer cells even before treatment begins due to the presence of multidrug resistance (MDR) gene [11]. Chemoresistance can also develop during treatments because of the activation of multiple signaling pathways [12, 13]. We hypothesized that COMP may mediate chemoresistance in breast cancer based on several previous findings. First, prostate cancer cells expressing COMP were resistant to apoptosis inducers and chemotherapeutic agents [2]. Second, COMP has the potential to inhibit apoptosis [14] by increasing the expression of apoptosis inhibitors in the PI3K/Akt pathway [15]. Higher COMP expression in breast cancer cells reduced ER stress, providing a survival advantage to tumor cells despite high levels of general protein production [14]. We also demonstrated that reducing endoplasmic reticulum (ER) stress resulted in the down-regulation of apoptosis in cancer cells expressing COMP [1], and this was achieved by preventing Ca^2+^ release from the ER of prostate cancer cells.

In this study, we investigated COMP-dependent resistance to chemotherapy in breast cancer and explored its complex molecular mechanisms. Tumors that developed in a mouse xenograft model, using COMP-expressing breast cancer cells, were resistant to doxorubicin treatment. In vitro, COMP-expressing breast cancer cell lines were resistant to chemotherapy, HER2-targeted therapy, and endocrine therapy. We demonstrated that the intracellular, ER-localized COMP confers resistance to treatment by interacting with and inactivating calpain. Breast cancer cells expressing COMP had disengaged caspases -9, -7, and -3, were protected from DNA damage and expressed higher levels of anti-apoptotic proteins, during chemotherapy treatment. Lastly, breast cancer patients with tumors expressing COMP, and undergoing chemotherapy or endocrine therapy, exhibited decreased overall survival (OS) in comparison with patients with tumors showing low COMP expression.

## Results

### Expression of COMP increases chemoresistance both in vivo and in vitro

**Fig. 1:**
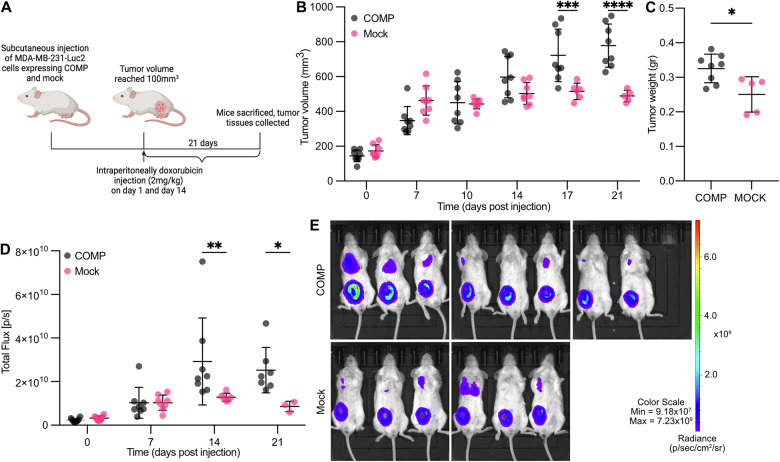
COMP expression promotes chemoresistance in orthotopic xenograft breast cancer tumor mouse model. **A** Schematic representation of the doxorubicin treatment of the NXG mouse model orthotopically transplanted with five million MDA-MB-231 Luc breast cancer cells. **B** Tumor volume measured by caliper during doxorubicin treatment. **C** Tumor weight at the end point of the experiment. **D** In vivo bioluminescence imaging of treated mice using the IVIS system. **E** IVIS images of doxorubicin-treated mice at day 14 post injection. Eight mice per group were included at the beginning. Statistical significance in **B** and **D** was calculated by two-way ANOVA with Sidak post-hoc test, while in **C** by Mann–Whitney test. *p < 0.05; **p < 0.01; ***p < 0.001 and ****p < 0.0001.

A xenograft orthotopic breast tumor model was established to assess whether COMP may promote chemoresistance in vivo (Fig. 1A). Mice were treated with 2 mg/kg of doxorubicin twice, with treatments administered 15 days apart, after tumors reached 100 mm³. At the experiment’s endpoint, the volume of the COMP-expressing tumors was 1.6 times larger (Fig. 1B), and there was significant difference in weight of the tumors and survival (Figs. 1C and S1). Interestingly, from day 14 after doxorubicin injection, the number of viable cancer cells in the COMP expressing tumors was twice as high as those in the mock control group, as examined by measurement of bioluminescence with the IVIS imaging system (Fig. 1D, E). These data illustrate that COMP expression by the breast cancer cells induces chemoresistance in vivo.

**Fig. 2:**
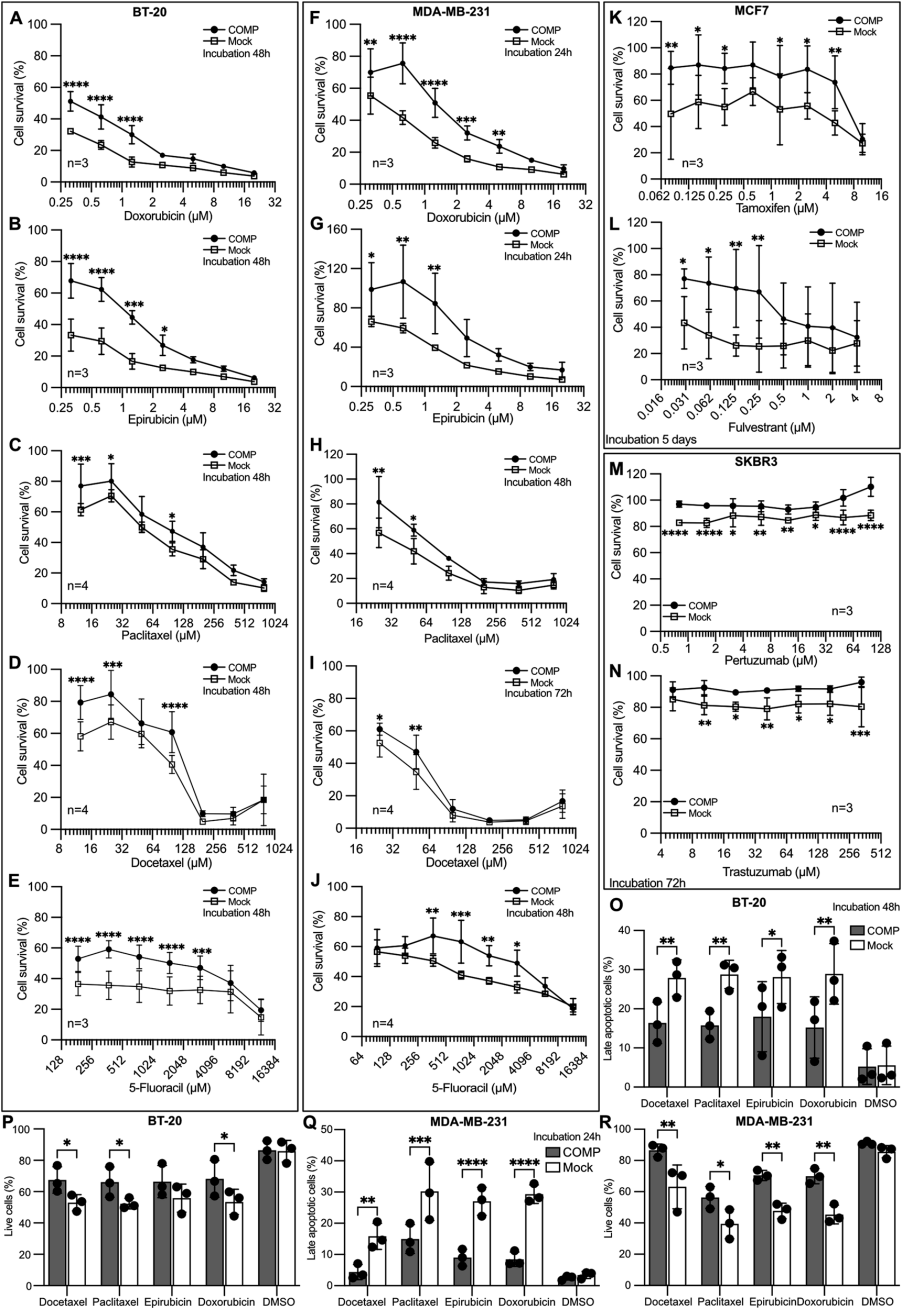
Breast cancer cells line expressing COMP are resistant to chemotherapeutic drugs, endocrine therapy and anti-HER2 therapy. Cyquant survival assay was performed using (**A**–**E**) BT-20 and (**F**–**J**) MDA-MB-231 cells expressing COMP and mock control treated with doxorubicin, epirubicin, paclitaxel, docetaxel, and 5-fluoracil, respectively, at the indicated concentrations. **K**, **L** Cyquant survival assay was performed in MCF-7 cells expressing COMP and respective mock control cells treated with tamoxifen and fulvestrant. **M**, **N** Cyquant survival assay was conducted in HER2-positive SKBR3 cells expressing COMP and mock control cells treated with pertuzumab and trastuzumab. Late apoptotic and live cells were determined by flow cytometry in both (**O**, **P**) BT-20 and (**Q**, **R**) MDA-MB 231 cells following anti-cancer chemotherapeutic agents treatment. Data are representative of at least three independent experiments. Statistical significances were determined by repeat measurements two-way ANOVA with Sidak post-hoc test. *p < 0.05; **p < 0.01; ***p < 0.001 and ****p < 0.0001.

To evaluate the effect of COMP on chemoresistance in vitro, a cell survival assay (Cyquant) was performed. The triple negative cell lines BT-20 and MDA-MB-231 expressing COMP or their mock control cells were treated with first-line chemotherapy drugs. The survival rate was higher in COMP-expressing cells than in mock cells when treated with doxorubicin, epirubicin, paclitaxel, docetaxel and 5-fluoracil in both cell lines (Fig. 2A–J). Additionally, estrogen receptor-positive breast cancer MCF7 cells expressing COMP were treated with the endocrine therapy drugs tamoxifen and fulvestrant. COMP-expressing cells were more resistant to these drugs compared to mock cells (Fig. 2K, L). Subsequently, we investigated the sensitivity of HER2-positive cells expressing COMP to anti-HER2 antibodies: pertuzumab and trastuzumab. COMP-expressing SKBR3 cells were more resistant to the treatment than mock cells (Fig. 2M, N). When the half-maximal inhibitory concentration (IC_50_) was calculated, we observed that a higher dose of the drugs was needed for breast cancer cell lines expressing COMP to achieve effect similar to the mock cells (Fig. S2). The percentages of apoptotic cells after treatment with several chemotherapeutic agents were higher in mock than in COMP-expressing cells when the annexin V-APC and zombie aqua dual staining assay was performed using flow cytometry (Figs. 2O–R and S3). These data confirm our observations obtained using the Cyquant assay. Collectively, both in vivo and in vitro data demonstrated that COMP expression promotes resistance to therapy in breast cancer cells.

### Intracellular but not secreted COMP renders cells chemoresistant

**Fig. 3:**
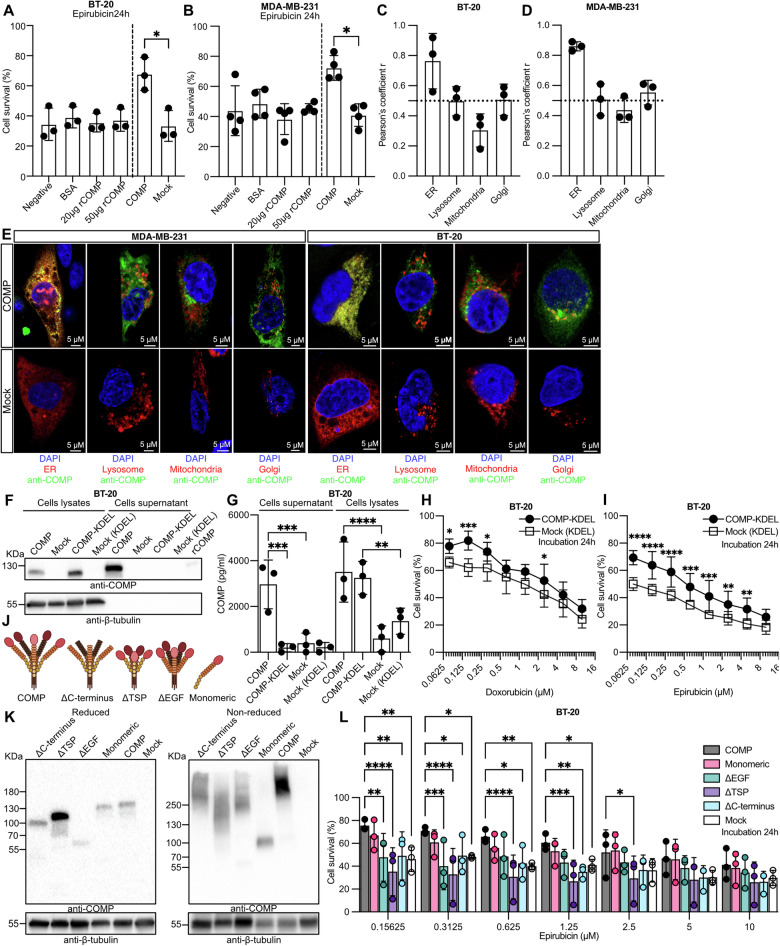
COMP chemoresistance mediated by the presence of COMP in the ER. Cyquant survival assay in BT-20 (**A**) and MDA MB-231 (**B**) wild-type cells supplemented with recombinant COMP (rCOMP) followed by treatment with 0.312 μM epirubicin for 24 h. Statistical significances were determined by one-way ANOVA with Tukey post-hoc test. Cells treated with BSA and epirubicin alone were used as negative control. Pearson’s coefficient r was calculated for COMP and various cells organelles with Fiji software from confocal microscope images captured from BT-20 (**C**) and MDA-MB-231 (**D**) cells stained for ER, lysosomes, mitochondria and Golgi apparatus. **E** Representative image of intracellular colocalization study of anti-COMP (pseudo colored green; ER: Alexa fluor 488, Lysosomes and Golgi: Alexa fluor 647, Mitochondria: Alexa fluor 546) in MDA-MB-231 and BT-20 cells expressing COMP and mock by confocal microscopy. DAPI (pseudo colored blue) was used to stain the nuclei; CellLight ER-RFP (pseudo colored red), was used to stain the ER; LysoTracker Red DND-99 (pseudo colored red) was used to stain the lysosomes; MitoTracker Deep Red (red) was used to stain the mitochondria; Primary antibody against GM130 and secondary conjugated with Alexa fluor 546 (pseudo colored red), was used to stain the Golgi apparatus. COMP protein expression in cell lysates and supernatants was assessed by (**F**) western blot and (**G**) ELISA in BT-20 cells expressing COMP, COMP-KDEL and mock control, respectively. Cyquant survival assay in BT-20 cells expressing COMP-KDEL treated with different concentrations of (**H**) doxorubicin and (**I**) epirubicin. **J** Graphic representation of the four COMP mutants in which was deleted: the EGF domains (ΔEGF), the TSP repeats (ΔTSP), the C-terminal region (ΔC-terminus), and the polymerization N-terminal region yielding monomeric COMP. (**K**) Expression of truncated COMP in BT-20 cells was verified by western blot analysis under reduced and non-reduced (absence of DTT in loading buffer) conditions. **L** Cyquant survival assay of BT-20 cells expressing COMP, COMP mutants and mock was performed after treatment with different concentrations of epirubicin. Data are representative of at least three independent experiments. Statistical significances were determined by repeat measurements two-way ANOVA with Sidak post-hoc test. *p < 0.05; **p < 0.01; ***p < 0.001 and ****p < 0.0001.

Since COMP is a secreted protein that interacts with the surrounding ECM but also rebinds to the surface of cancer cells, we queried whether the secreted COMP or the intracellular COMP protects the cancer cells from apoptosis. When incubated with purified recombinant COMP (rCOMP), the percentage of viable BT-20 (Fig. 3A) or MDA-MB-231 (Fig. 3B) cells was comparable to that of control cells treated with epirubicin alone. This indicates that extracellular COMP does not protect cells from death, and we therefor postulated that the intracellular COMP may be responsible for the observed chemoresistance. To assess the intracellular localization of COMP, we stained the endoplasmic reticulum (ER), lysosomes, mitochondria and Golgi apparatus of BT-20 and MDA-MB-231 cells with specific anti-COMP antibodies (Figs. 3C–E and S4). It was evident that COMP was mainly localized in the ER with high Pearson’s coefficient r (BT-20: r = 0.763, MDA-MB-231: r = 0.860). Moreover, COMP was partially colocalized with the lysosomes (BT-20: r = 0.497, MDA-MB-231: r = 0.507) and the Golgi (BT-20: r = 0.507 MDA-MB-231: r = 0.553). Consequently, we hypothesized that COMP, when localized intracellularly in the ER, rescues cancer cells from apoptosis. To assess this, we introduced the KDEL peptide just before the stop codon of the protein (COMP-KDEL; Fig. S5). The KDEL peptide prevents the protein from being exported from the ER and thus secreted, even when it carries a signal peptide in the N-terminus [16]. As predicted, COMP-KDEL was found only in the cell lysate but not in the supernatant, while wt COMP was readily secreted as shown by western blot and ELISA data (Fig. 3F, G). Strikingly, BT-20 cells expressing COMP-KDEL had higher survival rate compared to mock cells when treated with doxorubicin (Fig. 3H) and epirubicin (Fig. 3I), in a concentration-dependent manner. Accordingly, the calculated IC_50_ of COMP-KDEL expressing BT-20 cells was higher than the mock cells (Fig. S6). This finding supports the hypothesis that intracellular ER-localized COMP is responsible for the observed chemoresistance in cancer cells.

### TSP repeats are crucial for COMP induced chemoresistance

To gain insight into which domains of COMP are responsible for its effect on drug resistance, four COMP mutants were constructed by deleting the EGF domains (ΔEGF), the TSP repeats (ΔTSP), the C-terminal region (ΔC-terminus) and the polymerization N-terminal region yielding the monomeric COMP (Fig. 3J). The expression of COMP mutants in BT-20 cells was detected by western blot under reducing conditions. Additionally, the non-reducing conditions, confirmed that mutants were polymeric except for the ΔC-terminus COMP (Fig. 3K). A survival assay was used to assess the chemoresistance of cell lines expressing COMP mutants. Compared to the cells expressing full-length COMP, cell viability in ΔTSP, ΔEGF and Δ-terminus was decreased (Fig. 3L). Furthermore, the lowest IC_50_ value for ΔTSP was observed to be ∼0.067 μM compared to the IC_50_ of COMP ∼1.135 μM (Fig. S7). These data indicate that TSP repeats are crucial for the effect of COMP on chemoresistance.

### COMP affects intracellular calcium homeostasis

**Fig. 4:**
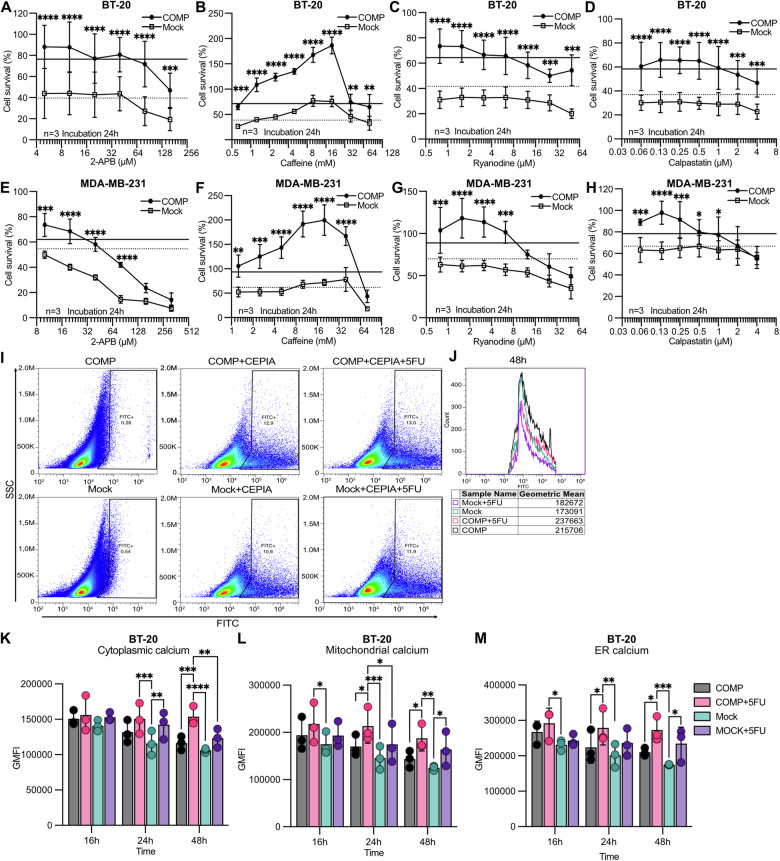
COMP influences intracellular calcium. Cyquant survival assay was performed in (**A**–**D**) BT-20 and (**E**–**H**) MDA-MB-231 cells treated with 0.625 μM epirubicin and various calcium inhibitors. Cells treated with epirubicin alone are shown as a continuous black line for COMP and discontinuous black line for mock. The anti-cancer drug 5-fluoracil (5FU) 750 μM was used to induce apoptosis, and the calcium levels were measured with genetic censors in the cytoplasm (pCase12-Cyto), mitochondria (pCase12-Mito), and ER (G-CEPIA). **I** Representative dot plots of the FACS gating strategy. **J**–**M** Quantification of the calcium levels by calculating the geometric mean fluorescence of gated cells at different time-points within the cytoplasm, mitochondria, and ER. Data are representative of at least three independent experiments. Statistical significances were determined by repeat measurements two-way ANOVA with Sidak post-hoc test. *p < 0.05; **p < 0.01; ***p < 0.001 and ****p < 0.0001.

ER not only contributes to protein synthesis but also serves as intracellular calcium storage. We assessed the potential mechanism by which ER-localized COMP renders cancer cells resistant to apoptosis by influencing the intracellular calcium homeostasis. Cell survival was evaluated under chemotherapy treatment in the presence of several intracellular calcium modulators. Calcium inhibitors such as 2-APB, caffeine, ryanodine, and calpastatin did not reverse the effect of COMP on chemoresistance (Figs. 4A–H and S8).

Next, we investigated the intracellular calcium levels within cellular organelles. We used the anti-cancer drug 5-fluoracil and measured calcium levels in cytosol (pCase12-Cyto), mitochondria (pCase12-Mito), and ER (G-CEPIA) using genetic censors. For every measurement 10,000 FITC positive cells expressing the genetic censor were assessed. During apoptosis, we observed extensive calcium influx into the cytoplasm, followed by calcium overload in the mitochondria and finally apoptosis (Fig. 4I, J). The cytosolic calcium level increased significantly in mock cells after 24 h but remained stable in COMP-expressing cells following the drug treatment (Fig. 4K). At 48 h post-treatment, the cytosolic calcium level was significantly higher in cells expressing COMP under chemotherapy than in mock cells. Additionally, mitochondrial calcium levels were elevated in cells expressing COMP when treated with 5-fluoracil at 24 h and 48 h compared to the untreated COMP-expressing cells, whereas mock-treated cells reached high mitochondrial calcium levels at 48 h (Fig. 4L). Further, we observed stable levels of increased ER calcium at 24 h and 48 h following 5-fluoracil treatment in cells expressing COMP (Fig. 4M). This result indicates that COMP affects intracellular calcium homeostasis but cannot explain the regulation of the apoptosis pathway in COMP cells after anti-cancer drug treatment.

### Expression of COMP protects cells from apoptosis

**Fig. 5:**
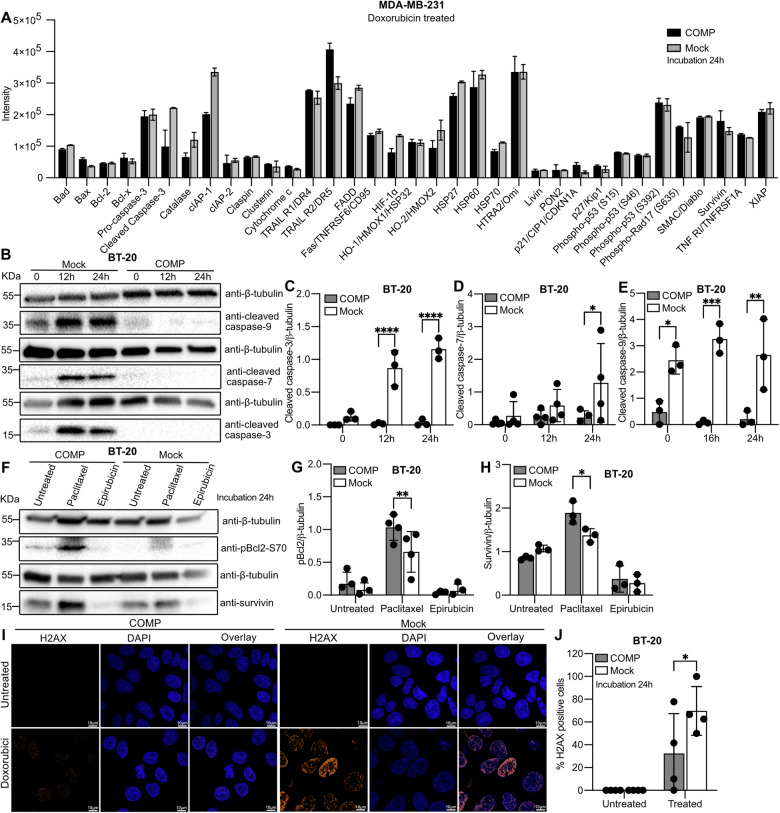
COMP-expressing breast cancer cells under chemotherapy exhibit less activated caspases and DNA damage. **A** Apoptosis antibody array using lysates from MDA-MB-231 cells expressing COMP and mock treated with 0.5 μM doxorubicin for 24 h. Samples represent technical replicates. **B** Cleaved caspase-3, -7 and -9 levels were assessed by western blot on BT-20 cells expressing COMP and mock after 10 μM epirubicin treatment. **C**–**E** Quantification of cleaved caspase-3, -7 and -9 intensity, using β-Tubulin as a housekeeping protein. **F** Levels of p-Bcl2 and survivin were assessed by western blot on BT-20 cells expressing COMP and mock after treatment with 10 μM paclitaxel or 10 μM epirubicin for 24 h. **G**, **H** Quantification of p-Bcl2 and survivin intensity, using β-Tubulin as a housekeeping protein. Representative image (**I**) and quantification (**J**) of DNA damage via γH2AX foci in COMP and mock cells treated with 1 μM doxorubicin. Data are representative of at least three independent experiments. Statistical significances were assessed by repeat measurements two-way ANOVA with Sidak post-hoc test. *p < 0.05; **p < 0.01; ***p < 0.001 and ****p < 0.0001.

To dissect the molecular mechanism through which COMP protects cells from death, we used an antibody expression array targeting apoptosis-related proteins. Doxorubicin was used to trigger apoptosis and three targets emerged, including cleaved caspase-3, catalase, and cIAP-1, all of which were all down-regulated in COMP-expressing cells compared to mock (Fig. 5A). The observation for caspase-3 was confirmed by western blot analysis of cleaved caspase-3 following epirubicin treatment (Fig. 5B, C). Additionally, western blot analysis of cleaved caspase-7 and -9 revealed a similar pattern to that of cleaved caspase-3 (Fig. 5D, E). All cleaved, i.e. activated caspases were significantly down-regulated, almost absent, in COMP-expressing cells compared to the mock cells under epirubicin treatment. In contrast, the caspase-8 activation was not detected when the cells were treated with epirubicin (Fig. S9). Furthermore, we also investigated the effect of COMP on the expression of anti-apoptotic proteins. Phospho-Bcl2 and survivin were both increased in COMP-expressing cells compared to mock when treated with paclitaxel drug (Fig. 5F–H). There were no significant changes in cells treated with epirubicin as reported previously [17]. No cytochrome C level variation was noticed despite the high p-Bcl2 in COMP expressing cells following paclitaxel therapy (Fig. S10).

### COMP protects cells from DNA damage

The DNA damage induced by chemotherapeutic drugs such as doxorubicin or epirubicin can lead to apoptosis. Subsequently, we investigated whether DNA damage is influenced by the expression of COMP. Doxorubicin was used since its mechanism of action is primarily to inhibit nuclear DNA transcription and replication while increasing DNA damage by inhibiting topoisomerase II activity. With a γ-H2AX dot corresponding to a DNA double-strand break [18], the result showed that there is less DNA damage in COMP-expressing cells than in mock (Fig. 5I, J). This finding indicates that COMP protects cells from DNA damage-induced apoptosis.

### COMP interacts with and inactivates calpain

**Fig. 6:**
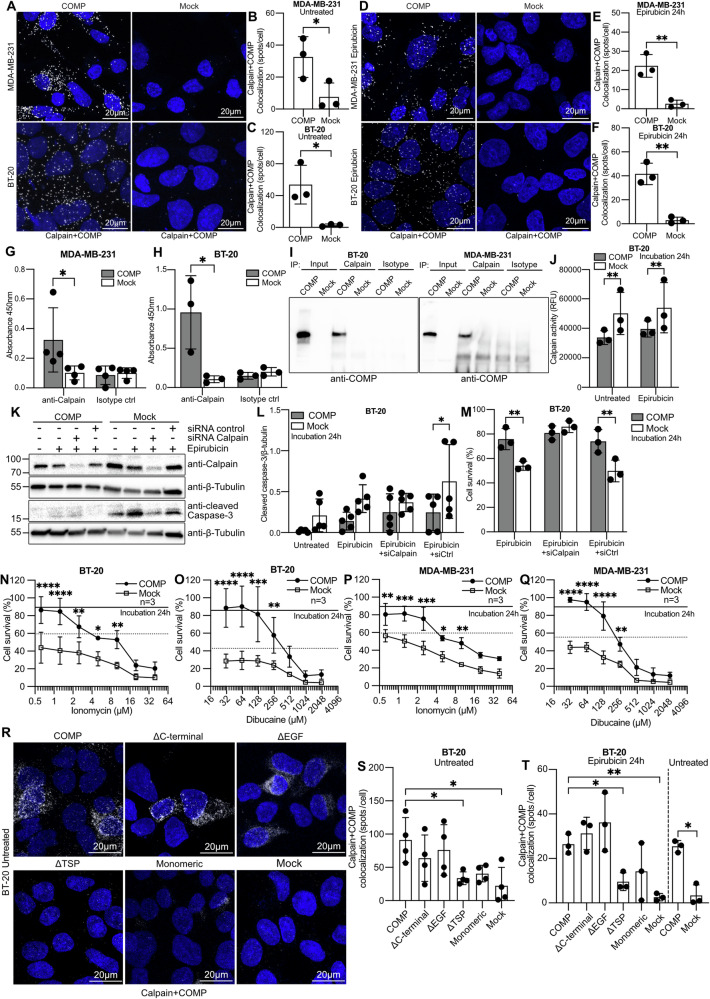
COMP binds through the TSP repeats and deactivates calpain leading to chemoresistance. **A** PLA assay in both BT-20 and MDA-MB-231 cells expressing COMP and mock. In the representative image, the white dot indicates the interaction of COMP and calpain. **B**, **C** Quantification of the dots per cell displaying the number of interactions between COMP and calpain. **D**–**F** Similar experiment was performed using 0.625 μM of epirubicin treatment for 24 h. Statistical significances were determined by t-test. ELISA assay was performed to evaluate the interaction of COMP and calpain in (**G**) MDA-MB-231 and (**H**) BT-20 cells. **I** Calpain was immunoprecipitated and COMP was detected with western blot analysis. Cell lysates sample was collected as a control of COMP expression in the input. Mouse IgG1antibody isotype was used as a negative control during the immunoprecipitation. **J** Fluorogenic calpain activity kit in BT-20 cells expressing COMP and mock. **K** Calpain silencing, with siRNA, abolished the apoptosis protection of COMP as shown by the cleaved caspase-3 level **L** Quantification of the cleaved caspase-3 level using β-Tubulin as a housekeeping protein. **M** Evaluation of cell survival with the Cyquant assay under 0.625 μM epirubicin treatment and calpain silencing, with siRNA for 24 h. Cyquant survival assay following the calpain activators ionomycin and dibucaine with 0.625 μM epirubicin treatment in (**N**, **O**) BT-20 and (**P**, **Q**) MDA-MB-231 cells. Cells treated with epirubicin alone were represented as continuous black line for COMP and discontinuous black line for mock. **R** Representative image of PLA assay in BT-20 cells expressing COMP, COMP mutants and mock. The white dot represents the interaction of COMP mutants and calpain in cells. **S** Quantification of the dots per cell showing the number of interactions between COMP mutants and calpain. Statistical significances were determined by one-way ANOVA with Dunnett post-hoc test. **T** similar experiment was performed with the BT-20 cells expressing the COMP mutants using 0.625 μM epirubicin treatment for 24 h. Data are representative of at least three independent experiments. Statistical significances were determined by repeat measurements two-way ANOVA with Sidak post-hoc test. *p < 0.05; **p < 0.01; ***p < 0.001 and ****p < 0.0001.

Calpains are calcium-dependent proteases linked to breast cancer cell proliferation and apoptosis [19]. The calpain family is classified into ubiquitous, tissue-specific, and atypical calpains [20]. There are two ubiquitous calpain isoenzyme forms: calpain I also known as μ-calpain and calpain II also known as m-calpain, and they are distinguished by their different intracellular calcium requirements. In our study, the term “calpain” refers to the ubiquitous calpain I. PLA assay indicated that COMP interacts with calpain in both MDA-MB-231 and BT-20 cell lines (Fig. 6A–C). The interaction between COMP and calpain was retained under epirubicin treatment for 24 h (Fig. 6D–F). This interaction was also confirmed using an ELISA assay (Fig. 6G, H) and co-immunoprecipitation (Fig. 6I). Meanwhile, cells expressing COMP had less calpain activity than mock cells under basal conditions and upon treatment with epirubicin (Fig. 6J). To assess the role of calpain in COMP-mediated chemoresistance, we successfully down-regulated calpain (Fig. S11). There was no significant difference in cleaved caspase-3 levels between COMP and mock control cells after epirubicin treatment when calpain was silenced (Fig. 6K, L). As expected, the difference in survival between COMP-expressing BT-20 cells and mock cells disappeared when calpain was silenced because the inhibition of calpain increases number of live mock cells, as is well documented (Fig. 6M). Interestingly, when calpain activators such as ionomycin and dibucaine were used together with the chemotherapeutic drug, the survival rate of cells expressing COMP was significantly reduced compared to the COMP cells treated with epirubicin alone (Figs. 6N, Q and S12). These results indicate that cells expressing COMP become more sensitive to treatment, implying that calpain activation attenuated COMP-mediated chemoresistance in breast cancer cells. Notably, the PLA assay between COMP mutants and calpain showed that COMP lacking TSP repeats does not interact with calpain (Fig. 6R, S). Similar results were obtained when BT-20 cells expressing the mutants were treated with epirubicin (Figs. 6T and S13). Furthermore, we checked the level of calpastatin, a potent calpain inhibitor [21], but no difference was detected among the untreated, epirubicin-treated and exogenous calpastatin treated COMP and mock cells (Fig. S14).

### Breast cancer patients undergoing therapy, and whose tumors expressed high levels of COMP exhibited decreased overall survival

**Fig. 7:**
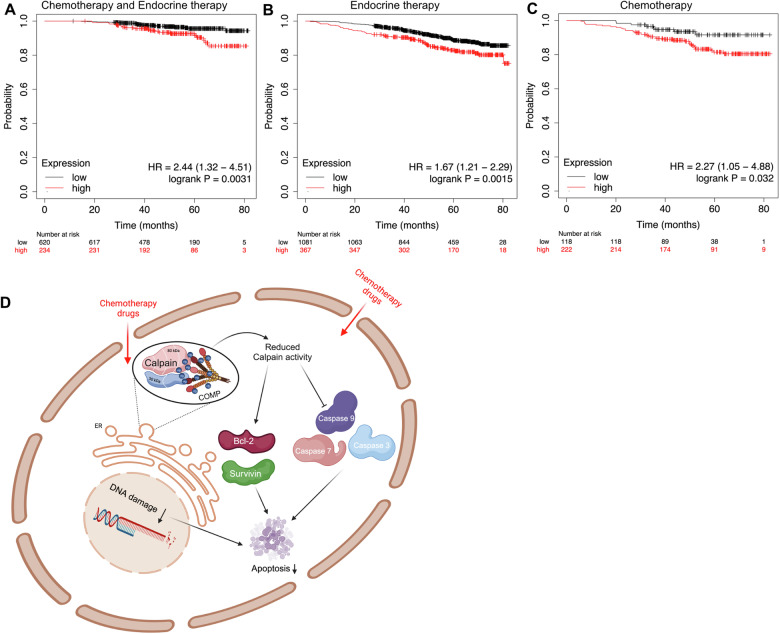
High COMP expression reduces overall survival in breast cancer patients under any type of therapy. Kaplan–Meier analysis was used to evaluate the OS from breast cancer patients with high or low COMP expression who received both (**A**) chemotherapy and endocrine therapy, (**B**) or endocrine therapy alone or (**C**) chemotherapy alone. **D** Graphical representation of the proposed molecular mechanism by which COMP induces resistance to apoptosis and cancer therapy, created with BioRender.com.

Onwards, we aimed to investigate the data of breast cancer patients who were undergoing antitumor therapy. We retrieved patients’ data from the Kaplan-Meier breast RNA-seq plotter online tool [22]. The Kaplan–Meier plotter is a manually curated database in which the gene expression data, the relapse free and overall survival are retrieved from GEO, EGA and TCGA databases. The patient samples were divided in two groups utilizing the best cutoff options of the Kaplan-Meier plotter. Breast cancer patients who received both endocrine therapy and chemotherapy had shorter OS (HR: 2.44, *p* = 0.0031) when they had tumors expressing high levels of COMP compared to patients with tumors expressing low levels of COMP (Fig. 7A). The same effect on OS was observed when COMP expression levels were evaluated in patients who received endocrine therapy alone (HR: 1.67, *p* = 0.0015; Fig. 7B) or chemotherapy alone (HR: 2.27, *p* = 0.032; Fig. 7C).

## Discussion

We previously reported that COMP confers resistance to apoptosis in prostate cancer cells [2], but the molecular mechanism responsible for this phenomenon has not been studied in depth. Herein, using in vivo and in vitro experimental approaches, we established that COMP promotes breast cancer cells resistance towards many chemotherapeutic drugs, HER2-targeted therapy, and endocrine therapy. The observed resistance to therapy seems to be caused by inhibition of apoptosis and induction of anti-apoptotic proteins. The intracellular localization of COMP mainly in the ER was critical for the observed phenomenon and was mediated by the calcium-binding TSP repeats of COMP. Furthermore, calpain was identified as a novel ligand of COMP, which is required for its resistance to therapy.

Tumor growth in vivo, and in vitro assays measuring cell survival and apoptosis demonstrated resistance of COMP-expressing breast cancer cells against all studied types of anti-cancer drugs, which are used in clinics as a first or second line of therapy [23]. Chemotherapy taxanes (paclitaxel and docetaxel) were particular focus. These drugs, isolated from plants, act by promoting the polymerization of microtubules, leading to the formation of dysfunctional microtubules that hinder normal cellular processes and the cell cycle [24–26]. Similarly, anthracyclines (doxorubicin and epirubicin), which were isolated from *Streptomyces peucetius*, belong to a category of drugs that inhibit the synthesis of nucleic acids through DNA intercalation, oxidative stress induction, and topoisomerase II poisoning [27–29]. Fluoropyrimidines (5-Fluorouracil and capecitabine) were developed in the 1950s following the observation that hepatoma cells preferentially utilized the pyrimidine uracil compared to normal cells [30]. Their mechanism of action is attributed to the misincorporation of fluoronucleotides into RNA and DNA and the dysfunction of thymidylate synthase [31, 32]. For estrogen receptor-positive breast tumors, tamoxifen and fulvestrant were examined. Both drugs target the estrogen receptor; tamoxifen acts as an antagonist of estrogen, while fulvestrant induces the degradation of the receptor [33]. Lastly, for HER2-positive breast tumors, the monoclonal antibodies targeting the HER2 receptor, pertuzumab and trastuzumab, were used. Pertuzumab prevents HER2-mediated signaling and induces cytotoxicity, while trastuzumab inhibits the heterodimerization of HER2 with other HER family members, leading to the inhibition of downstream signaling [34]. Remarkably, cancer cells expressing COMP exhibited resistance to all the aforementioned anticancer therapies, regardless of their different mechanisms of action. Thus, we hypothesize that either the presence of COMP affects a fundamental cellular process, such as the previously demonstrated inhibition of calcium release from the ER in the context of prostate cancer [2], or multiple anti-apoptotic cellular mechanisms are impacted. In the current study, we did not examine experimentally effect of COMP on combination therapy. However, breast cancer patients treated in the clinic with various therapies and expressing high levels of COMP in tumors had worse overall survival. This indicates that COMP expressing tumors are resistant to many types of therapy currently used in the clinic including the combination therapy.

In this study, we discovered that the ER localized COMP induces chemoresistance as established using the COMP-KDEL model. The KDEL peptide is a well studies tag, which retains proteins in the ER and if they are leaked to any other organelle they are sorted back to the ER [35, 36]. Furthermore, extracellularly provided recombinant COMP was not able to confer resistance against chemotherapy. This observations align with the initial publication on COMP expression in breast cancer, which determined that COMP expression by the cancer cells correlated with a worse prognosis for breast cancer patients and could serve as an independent survival prognostic marker [1]. However, in the context of other types of cancer, such as colon cancer [4] and a certain type of pancreatic cancer [3], both the expression of COMP by the cancer cells and stroma were linked to a poorer overall survival of patients. It is possible that in these cancer types, the expression of COMP in the stroma is of greater significance. Additionally, in cancers where disease progression is heavily influenced by the tumor microenvironment, such as pancreatic cancer, COMP deposition in the extracellular matrix might affect the immune system, rendering the tumor more resistant to various therapies. Indeed, we have previously demonstrated that the presence of COMP in the tumor microenvironment correlates with tumor fibrosis, which inhibits the infiltration of CD8-positive T-cells into the tumor [3, 4].

Apoptosis is principally controlled by cysteine proteases, caspases. Caspases can be classified into two main categories, the initiator (Caspase-2, -8, -9 and -10), which respond to pro-apoptotic signals and activate the effector (or executioner) caspases (Caspase-3, -6 and -7). The effector caspases cleave numerus cellular targets resulting in morphological and biochemical transformations distinctive for apoptosis, such as DNA fragmentation and degradation of crucial cellular proteins [37]. Apoptosis can be initiated through two distinct mechanisms, the extrinsic pathway initiated by extracellular signals such as TNF (tumor necrosis factor) receptor stimulation by TNF-α, which leads to the formation of the death-inducing signaling complex (DISC). Within DISC the procaspase-8 or −10 are activated, which can then cleave the effector caspases. The intrinsic mitochondrial pathway is triggered by intracellular events such as ER stress, which shifts the balance of the Bcl-2 family of apoptotic proteins towards mitochondrial outer membrane permeabilization resulting in the release of cytochrome C, the formation of the apoptosome and the activation of caspase-9, which in turn activates the executioner caspases [38]. We were unable to detect any activation of the caspase-8 in COMP expressing cells treated with chemotherapy drugs. Tamoxifen and fulvestrant can potentially activate the extrinsic pathway. Cells expressing COMP showed resistance against apoptosis induced by both drugs. Moreover, when prostate cancer cells expressing COMP were treated with TNF-α, they exhibited protection against apoptosis. Thus, the effect of COMP in the extrinsic apoptosis pathway has to be further investigated in following studies.

Distinct inactivation of caspase-9 was observed in breast cancer cells expressing COMP in comparison to the control cells. Thus, we hypothesize that apoptosis in COMP-expressing cells is regulated via the intrinsic pathway. In contrast, the levels of cytochrome C remained stable during chemotherapy treatment potentially indicating intact mitochondria. Surprisingly, we discovered that COMP interacts with calpain. The importance of this interaction is supported by the fact that calpain has been reported in the ER, where it cleaves and activates initiator caspases of the intrinsic pathway [39]. Calpains are calcium-dependent cysteine proteases that can cleave various cellular substrates upon activation [40]. In line with this, we observed that activation of calpain was decreased in COMP-expressing cells. Previous reports have indicated that calpain activation can induce caspase-9 cleavage and activation, ultimately leading to apoptosis [41, 42]. Moreover, calpain silencing sensitized COMP-expressing cells to chemotherapy. These results are in line with previous studies, reporting that calpain knockdown induced apoptosis and impaired chemoresistance in lung cancer cells [43]. Therefore, we propose that COMP present in the ER inactivates calpain, resulting in apoptosis inhibition and enhanced chemoresistance (Fig. 7D). Furthermore, calpain activity can influence several cellular functions such as autophagy, cytoskeletal remodeling, migration, and cell cycle regulation.

To further dissect the molecular mechanism, mutants of COMP were constructed. The most significant finding was obtained by the deletion of the TSP repeats that comprise most of the calcium binding sites found in COMP. This deletion abrogated the interaction between COMP and calpain [14, 44]. It has been shown previously that mutations in the TSP repeats may have no effect on the folding and stability of COMP, but they do affect the number of calcium ions binding to COMP [45]. Thus, we hypothesize that the interaction between COMP and calpain is facilitated by the calcium ions bound to COMP. This hypothesis is further supported by the fact that calpain activity is highly regulated by the concentration of calcium ions. Undoubtedly, COMP chemoresistance is not due to one phenomenon, with several mechanisms likely involved. It was previously described that the resistance to apoptosis in COMP expressing cells could be due to the increase of apoptosis inhibitors [46]. In agreement with this observation, we found that anti-apoptotic proteins phospho-Bcl2 and survivin were increased in COMP-expressing cells upon chemotherapy.

The current study established that breast cancer cells expressing COMP were resistant to most first-line chemotherapy drugs, the HER-2-targeted drugs, and endocrine therapy drugs. The underlying molecular mechanism has been partially elucidated. Calpain interacts with COMP, leading to its inactivation, which inhibits the cleavage of caspase-9, -3 and -7 and induces the expression of anti-apoptotic proteins.

## Materials and methods

### Cell culture and transfection

The breast cancer cell lines BT-20, MCF-7, and MDA-MB-231 were purchased from American Type Culture Collection (ATCC, Virginia, USA). The SKBR3 cell line was obtained from Leibniz Institute, DSMZ-German Collection of Microorganisms and Cell Cultures (Germany). The cell lines BT-20 and MDA-MB-231 were cultured in high glucose DMEM medium (HyClone, Belgium), the SKBR-3 was cultured in RPMI 1640 (Hyclone) and the MCF-7 in low glucose DMEM supplemented with MEM non-essential amino acids (Gibco, Montana, USA) and 0.01 mg/ml human recombinant insulin (Sigma, Sweden). All media contained 10% fetal bovine serum (FBS) (ATCC), 1% penicillin/streptomycin (Hycult, Sweden). COMP expressing cells were generated as previously described [1]. Briefly, the transfections were performed in 6-wells plates when cells reached 75-85% confluence. Full-length COMP (human cDNA cloned in pCDNA3) or mock (empty pcDNA3 vector; Supplementary Table 1) were transfected into all cell lines using Lipofectamine 3000 (Thermo Fisher Scientific, Sweden) according to manufacturer’s instructions. Stable clones were selected after selection with 0.7 mg/ml geneticin (Thermo Fisher Scientific). The expression of COMP in the stably transfected cells was verified by western blotting as previously described [2]. Throughout the manuscript, cells expressing COMP are denoted as COMP, whereas mock cells transfected with empty pcDNA3 vector were used as a negative control. All cells were *mycoplasma* negative and tested bi-monthly externally by Eurofins genomics.

### Xenograft mouse model

Animal experimentation was conducted in accordance with ethical regulations and approved by the ethical committee of animal care of Lund University (9349/2020). Female NXG mice (NOD-*Prkdc*^*scid*^*-IL2rg*^*Tm1*^/Rj) aged at 14 weeks were injected with 5 × 10^6^ MDA-MB-231-Luc2 cells expressing COMP or control mock cells. The MDA-MB-231-Luc2 cell line was provided by TM (Faculty of Medicine, Saitama Medical University, Japan). Mice were orthotopically injected into the 4th left mammary fat pad. When the tumor volumes reached ~100 mm^3^, the treatment with doxorubicin (2 mg/kg) was initiated via intraperitoneal injection, on day 1 and day 14. All animals were included in analysis and were randomly assigned to control and treatment groups. Blinding was not performed in this study. Subsequently, tumor volume and body weight of the mice were monitored, and the tumors were imaged using IVIS Lumina Series III (PerkinElmer- Revvity) optical imaging. At the end point of the experiment, tumors were collected and weighed.

### Survival assay

Cells survival was measured using Cyquant assay (Thermo Fisher Scientific) according to manufacturer’s instructions. In brief, equal volume to culture media of 2xCyquant detection reagent was added to 96-well black-plates (Corning), incubated for 1 h, and the absorbance was measured by Cytation 5 (Biotek) at 450/535 nm. The percentage of viable cells was calculated using the following equation: viable cells (%) = absorbance of treated cells × 100/absorbance of untreated cells. BT-20 and MDA-MB-231 cells expressing COMP, or mutants of the protein, and their respective control mock cells were treated with chemotherapeutic drugs such as doxorubicin, epirubicin, docetaxel, paclitaxel, and 5-fluoracil, all from Selleckchem at concentrations and incubation times as indicated in figures. Moreover, various ER calcium inhibitors such as 2-aminoethyl diphenylborinate (2-APB) (Sigma), caffeine (TOCRIS), ryanodine (Calbiochem) and Acetyl-Calpastatin (TOCRIS) as well as calcium activators such as ionomycin (Sigma) and dibucaine (Sigma) at different concentrations indicated in the figures were tested in the presence of 0.625 μM epirubicin. The concentration of epirubicin was selected as the minimum drug concentration that produced the largest difference in the number of live cells between COMP-expressing and mock control cells when evaluated with the Cyquant assay.

For hormonotherapy the MCF-7 cells were seeded and incubated for 48 h, followed by replacing the medium with DMEM low glucose phenol red free (Gibco) supplemented with MEM non-essential amino acids and 0.01 mg/ml human recombinant insulin and containing 10% FBS Charcoal striped (Gibco), to deplete the estrogens. After 16 h, the medium was replaced with the same medium containing β-Estradiol (100 μM), tamoxifen (Sigma) and fulvestrant (Selleckchem) at concentrations indicated in figures. The medium was renewed after 3 days incubation and finally the surviving cells was measured after total 5 days of treatment. The HER2 positive SKBR3 cells expressing COMP and mock were treated with pertuzumab (Selleckchem) and trastuzumab (Selleckchem) for 72 h at concentrations showed in the figures. Furthermore, BT-20 cells were supplemented with purified recombinant COMP (20 ng and 50 ng) or BSA (50 ng) and treated with epirubicin (0.312 μM).

### Apoptosis assay

BT-20 and MDA-MB-231 COMP or mock cells were cultured in a 6-well plate. The cells were treated with chemotherapeutic drugs including docetaxel (20 μM), doxorubicin (0.156 μM), paclitaxel (10 μM) and epirubicin (0.156 μM). Apoptosis assay was performed using Annexin V-APC (Immunotools) and Zombie aqua (Biolegend) dual staining to distinguish live, apoptotic, and necrotic cells. The cells were washed with PBS, detached with trypsin, washed with FACS buffer (10 mM HEPES, 140 mM NaCl, 5 mM KCl, 1 mM MgCl_2_, 2 mM CaCl_2_, 0.02% NaN_3_), and incubated in the dark for 30 min in FACS buffer containing Annexin V-APC and Zombie aqua. Stained cells were washed with FACS buffer and data collected using Cytoflex flow cytometer (Beckman) and was analyzed with FlowJo software (BD).

### Localization of COMP in cellular organelles

Cells were seeded in 12-wells removable chamber slide (Ibidi) to reach 70% confluency the next day. Then, for ER visualization the CellLight ER-RFP, BacMam 2.0 (Thermo Fisher Scientific) was used to transfect the cells for 24 h according to manufacturer instructions, which led to the florescence staining of the ER with the RFP protein. For lysosome and mitochondria visualization cells were washed with media after 24 h and incubated with 75 nM of LysoTracker Red DND-99 (Thermo Fisher Scientific) and 250 nM of MitoTracker Deep Red (Thermo Fisher Scientific) for 1 h. For COMP and Golgi apparatus staining cells were fixed with 4% PFA in PBS and permeabilized with PBS with 0.1% Triton X-100, blocked with 3% BSA diluted in PBS with 0.1% Triton X-100 and were incubated with primary antibody against COMP and GM130 (Supplementary Table 3) at 4 °C overnight. Cells washed with PBS with 0.1% Triton X-100 and incubated with the appropriate secondary antibody (Supplementary Table 3), mounted with ProLong diamond antifade mounting medium with DAPI (Thermo Fisher Scientific) and visualized with ZEISS LSM 800 with Airyscan. Pearson’s coefficient r was calculated for COMP and various cells organelles with Fiji software.

### Construction of the ER-restricted COMP protein and COMP deletion mutants

To express ER- restricted and not secreted COMP, the KDEL peptide (Lys-Asp-Glu-Leu) was introduced in the C-terminus of COMP before the stop codon, the signal peptide in the N-terminus was retained. The COMP-KDEL, ΔTSP, and monomeric COMP were constructed utilizing modified protocols of the Quickchange Mutagenesis Kit for the addition or deletion of sequences (Agilent) [47]. The ΔC-terminus and ΔEGF were constructed by splicing through overlap extension PCR. Both methods were performed using specific mutagenesis primers (Supplementary Table 2), and the following protein domains were deleted: globular C-terminus region (ΔC-terminus), all EGF domains (ΔEGF), all TSP repeats (ΔTSP), and the N-terminal polymerization region, yielding a monomeric version of COMP. In all mutans the signal peptide in the N-terminus was retained. The generated plasmids carrying the mutated proteins were sequenced by Eurofins Genomics and the correct sequence was confirmed with DNASTAR software.

For the evaluation of the COMP-KDEL mutant pattern of expression an ELISA assay was performed. For collection of cells supernatant, the BT-20 cells were incubated for two days in opti-MEM medium (Gibco), the supernatants were collected and were concentrated by Amicon Ultra-15 centrifugal filters (Millipore-Merck, Germany). For collection of cell lysates, the cells were lysed with RIPA buffer (25 mM Tris-HCl pH 7.6, 150 mM NaCl, 1% Triton X-100, 1% Na-deoxycholate, 0.1% SDS) supplemented with 1% Halt protease and phosphatase inhibitors cocktail (Thermo Fisher Scientific). Then COMP expression in the cells lysates and supernatants were evaluated with Human COMP ELISA kit (R&D systems, #DY3134) according to the manufacturer’s protocol.

### Western blotting analysis

Cells were lysed with RIPA buffer prepared as mentioned earlier. For cell supernatant, cells were incubated for two days in opti-MEM medium, the medium was collected and concentrated by Amicom Ultra-15 centrifugal filters. Protein concentration was determined using BCA Protein Assay Kit (Pierce), equal number of proteins were separated on 4-15% Precast Protein gels (Bio-Rad) under reducing or non-reducing (absence of DTT in loading buffer) conditions and transferred onto PVDF membrane using Trans-Blot Turbo Transfer kit (Bio-rad), then blocked with 5% milk diluted in immunowash buffer (50 mM Tris-HCl pH 8.0, 150 nM NaCl, 0.05% Tween 20). Membranes were incubated overnight with the primary antibody (Supplementary Table 3) followed by incubation for 1 h at room temperature with the appropriate secondary HRP-conjugated antibody (Supplementary Table 3) and the signal was detected on the Bio-rad chemidoc system after incubating with ECL reagent (Millipore).

### Immunoprecipitation

BT-20 or MDA-MB-231 cells were seeded in 25 cm^2^ flasks to reach 70% confluency the next day. Then cells were lysed with NP-40 buffer (150 mM NaCl, 50 mM Tris-HCl [pH 7.5], 1% NP-40) and lysates containing the same amount of protein were incubated overnight with 5 μg anti-calpain mouse antibody (Supplementary Table 3). The next day 50 μl of protein G Dynabeads (Thermo Fisher Scientific) were added to precipitate the protein complexes. The beads three times washed three times with NP-40 buffer and the precipitated proteins were eluted with loading buffer at 95 °C and separated by electrophoresis.

### Proteome profiling

Human apoptosis array kit (R&D, #ARY009) was used to determine the relative levels of human apoptosis-related proteins, according to manufacturer instructions. Lysates from MDA-MB-231 cells expressing COMP and mock were used. For apoptosis induction cells were treated with doxorubicin (0.5 μM) for 24 h.

### DNA damage

Cells were seeded in 8-wells removable chamber slide (Ibidi) and treated with doxorubicin (1 μM) for 24 h to induce DNA damage. The slide was fixed with 4% PFA, permeabilized with PBS containing 0.1% Triton X-100, blocked with PBS containing 0.1% Triton X-100 and 3% BSA for 1 h at room temperature, then incubated overnight at 4 °C with phospho-H2AX antibody (Abcam). Cells were washed with PBS containing 0.1% Triton X-100 and incubated with Alexa fluor 546 goat anti-rabbit (Supplementary Table 3) for 1 h at room temperature. The cells were mounted with DAPI (Sigma) and visualized with confocal microscope ZEISS LSM 800. Cells with 10 or more H2AX foci were scored as positive. Number of H2AX foci were calculated with the Image J software.

### Intracellular calcium analysis

BT-20 cells expressing COMP or mock control cells were transiently transfected with the following plasmids using Lipofectamine 3000 (Thermo Fisher Scientific): pCMV G-CEPIA1er [48], a gift from Dr Masamitsu Iino (RRID: Addgene_58215), which was used to measure ER calcium, pCase12-Mito (Evrogen, Russia) for mitochondrial calcium and pCase12-Cyto for cytoplasmic calcium (Evrogen). The next day, the medium was replaced, and the cells were allowed to recover for 24 h, then treated with 5-fluoracil (750 μM) and incubated for different time points (16 h, 24 h and 48 h). For flow cytometry analyses, a sample of 10,000 untransfected cells was used to gate the genetic sensor expressing cells (FITC positive cells). Then every sample was analyzed by FACS until 10,000 genetic sensor expressing cells (FITC positive cells) were measured. Data were analyzed using FlowJo software.

### COMP interaction with calpain detected using Proximity ligation assay (PLA)

The PLA assay was performed utilizing the Duolink kit (Merck) according to the manufacturer’s instructions. Briefly, cells were seeded into 12-well removable chamber slides to reach 70% confluency the next day. Cells were fixed with 4% PFA in PBS, blocked with 3% BSA diluted in PBS with 0.1% Triton X-100, and incubated with primary antibodies (Supplementary Table 3) for 1 h at room temperature. Cells were washed with PBS containing 0.1% Triton X-100, followed by 1 h incubation with the appropriate probe (Merck) at room temperature. Cells were then washed with PBS including 0.1% Triton X-100, followed by DNA fragment ligation and amplification. Finally, cells were mounted with DAPI, visualized with ZEISS LSM 800 with Airyscan, and analyzed with ImageJ software.

### Calpain knock-down

In a 12-well plate 2 × 10^5^ of BT-20 COMP and mock cells were seeded and incubated overnight to attach. The cells were washed with PBS, and media replaced with OptiMem containing 20 nM of calpain siRNA SMARTpool mixture of 4 individual siRNAs (Dharmacon, L-005799-00-0005) was added in the Calpain silenced well and 100 nM of negative siRNA (Dharmacon, D-001810-02-05) in the negative control. After incubation for 24 h, the plate was washed and 0.625 μM of epirubicin was added following by another 24 h incubation. The cells were either analyzed with qPCR and western blot for successful calpain silencing or number of viable cells evaluated with Cyquant assay.

### Statistical analyses

All the experiments were performed in at least 3 independent biological repeats and are presented as the mean with standard deviation (SD). Prism 9 was used for the statistical analyses. When two groups were compared Student’s t-test was used. When more than two groups were compared for one variable one-way ANOVA test was used. When multiple groups were compared for more than two variables the two-way ANOVA was applied. Statistical test for repeat measurements was used when was appropriate for the experimental design. G-power software was used to calculate the effect size. P ≤ 0.05 was considered statistically significant.

### Supplementary information

Western blot full length uncropped original version

Supplementary data

## Data Availability

The datasets analyzed during the current study are available in the Kaplan-Meier Plotter.
