# Supplementary material for: Intracellular cartilage oligomeric matrix protein augments breast cancer resistance to chemotherapy
Source: Cell Death Dis. 2024 Jul 4;15(7):480. doi: 10.1038/s41419-024-06872-7 (PMC11224260; doi:10.1038/s41419-024-06872-7)

Western blot full length uncropped original version

Figure 3F

Anti-COMP

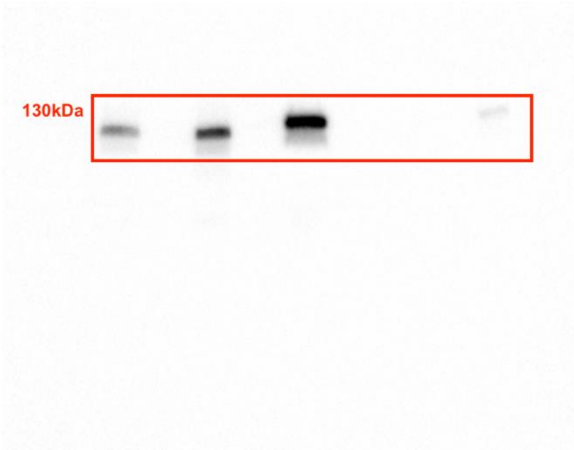

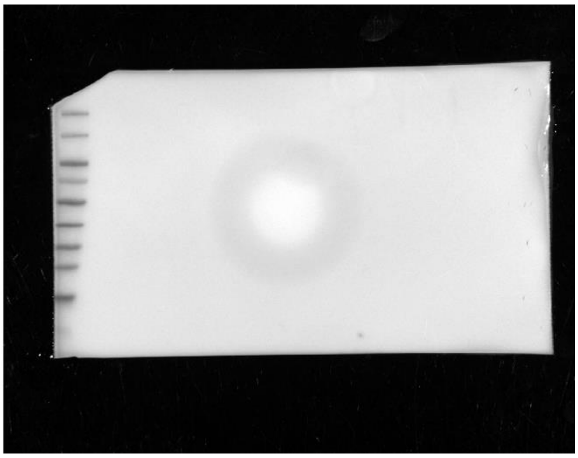

Anti-β-Tubulin

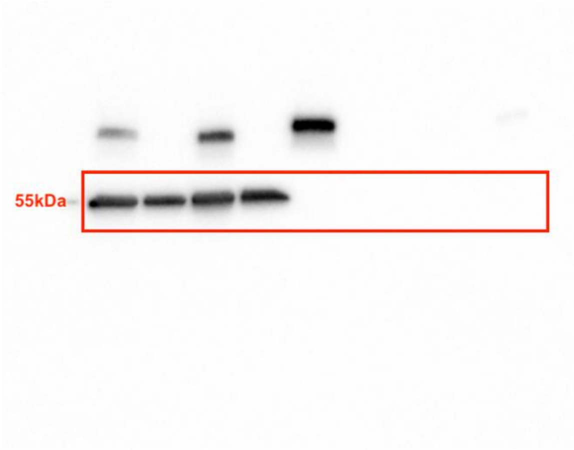

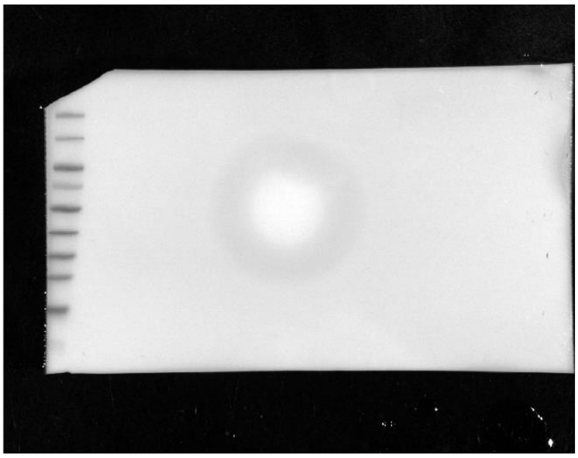

Figure 3K
Reduced

Anti-COMP

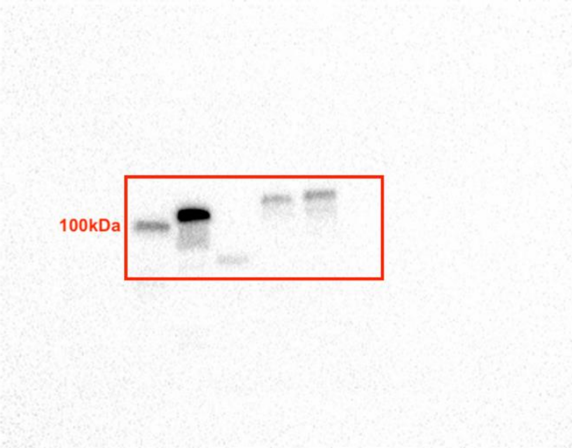

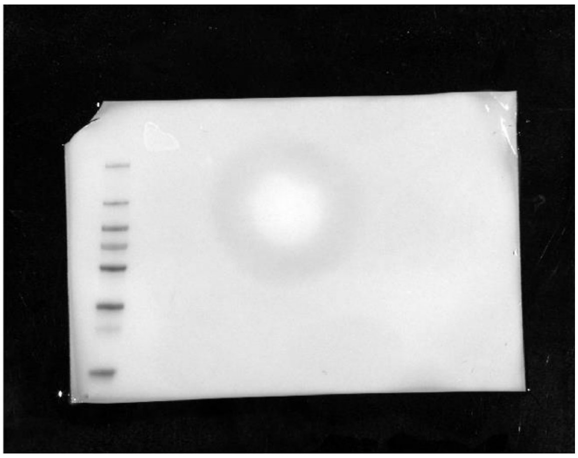

Anti-β-Tubulin

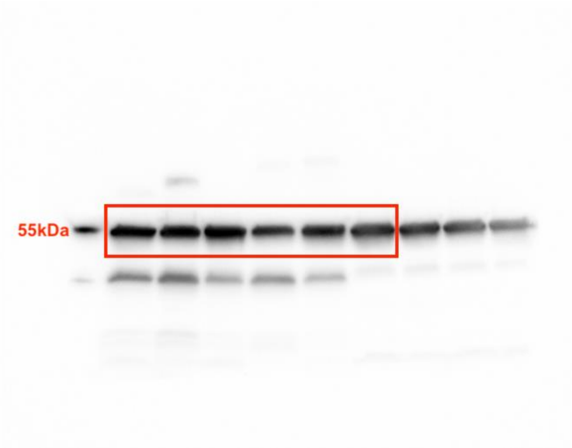

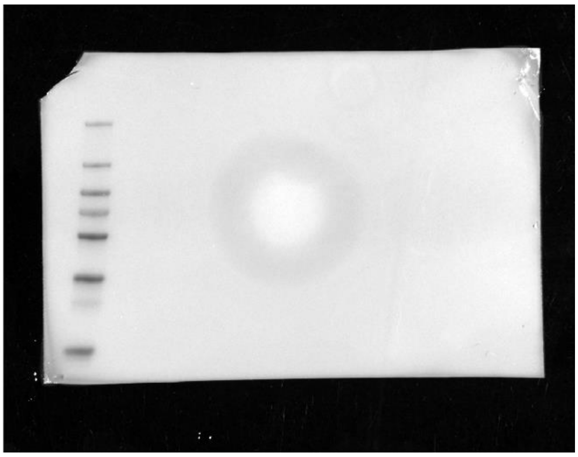

Non-reduced

Anti-COMP

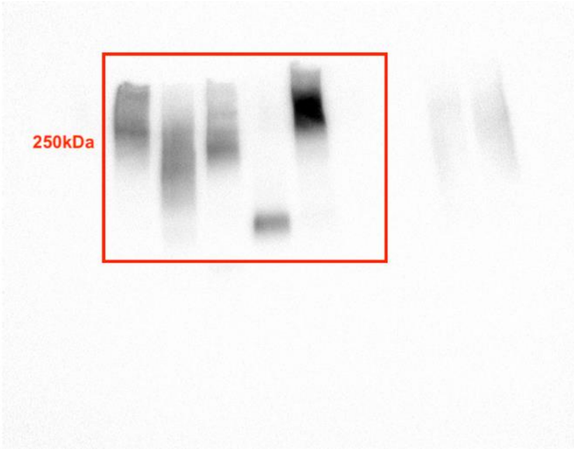

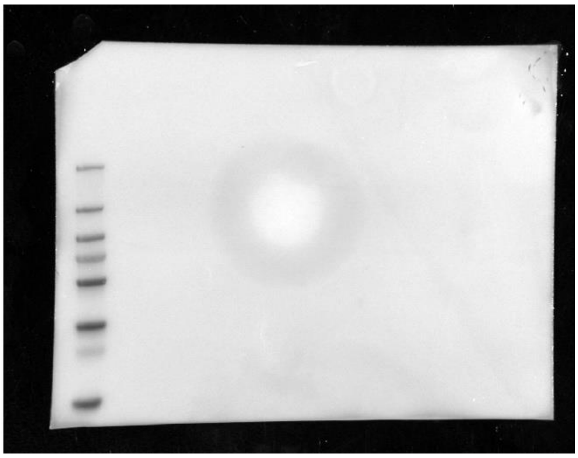

Anti-β-Tubulin

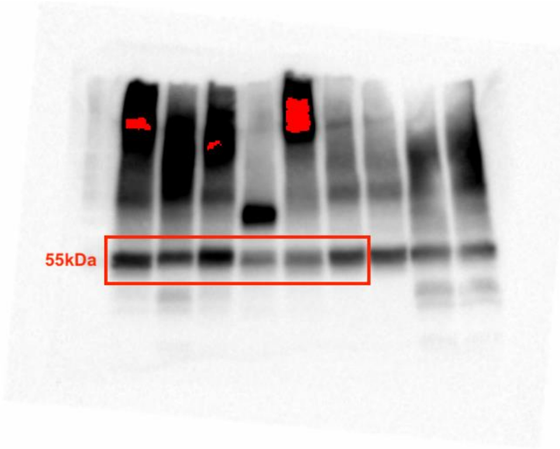

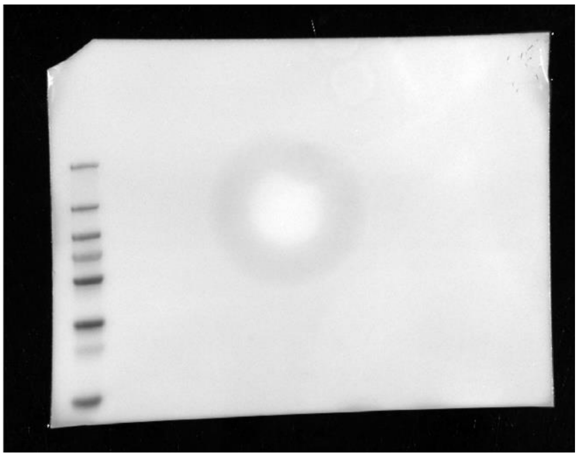

Figure 5B

Anti-β-Tubulin

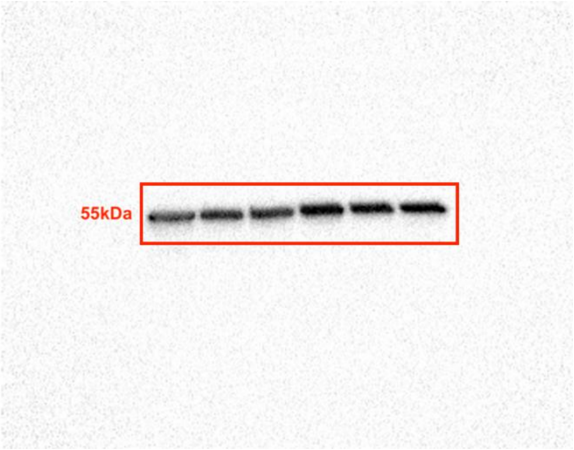

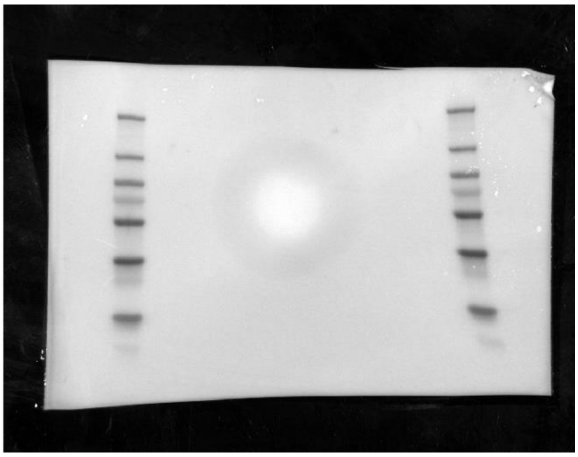

Anti-cleaved caspase 9

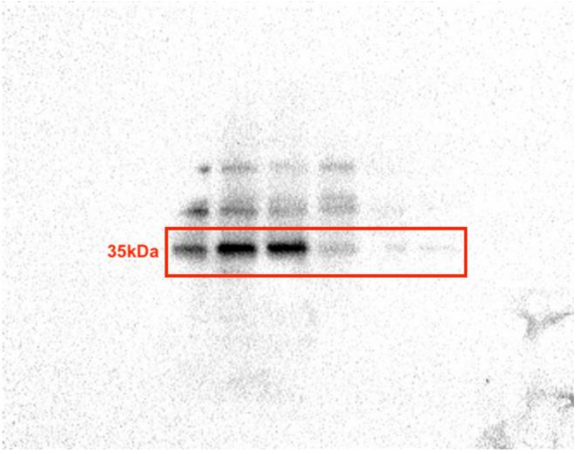

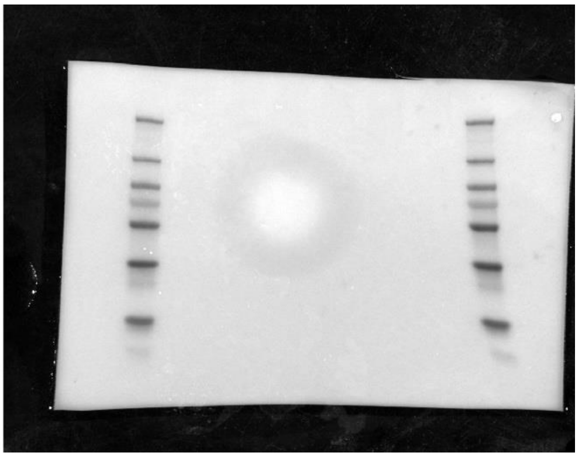

Anti-β-Tubulin

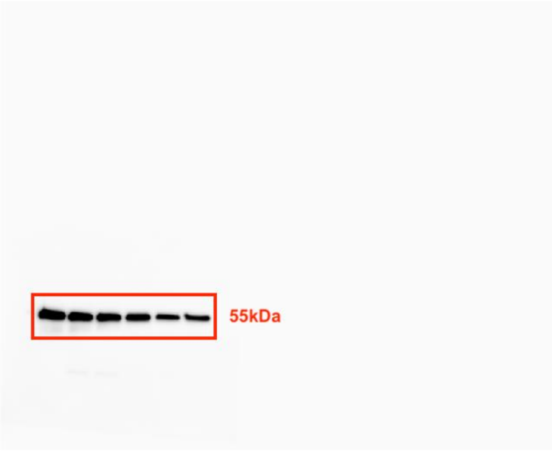

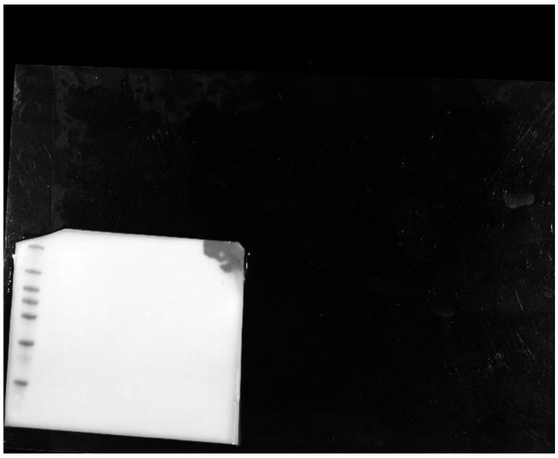

Anti-cleaved caspase 7

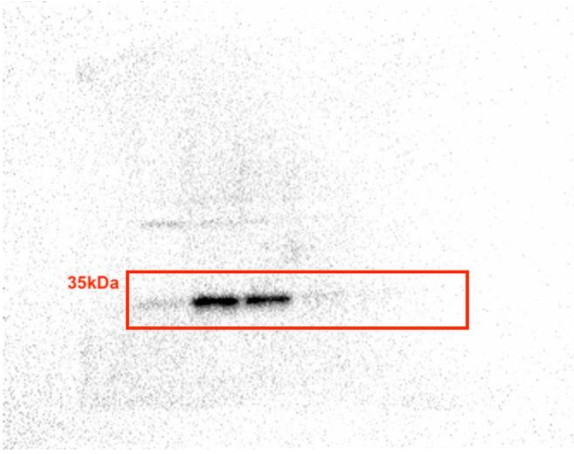

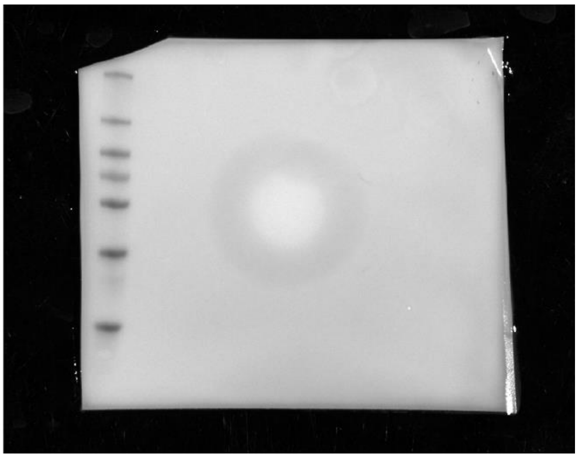

Anti-β-Tubulin

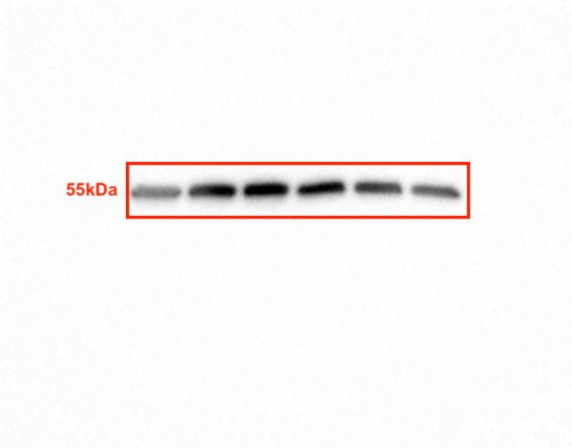

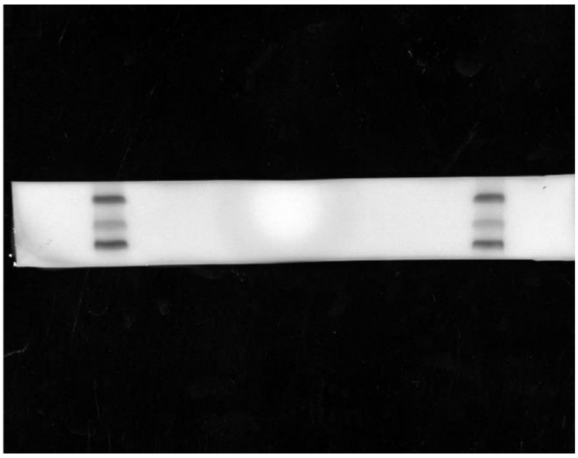

Anti-cleaved caspase 3

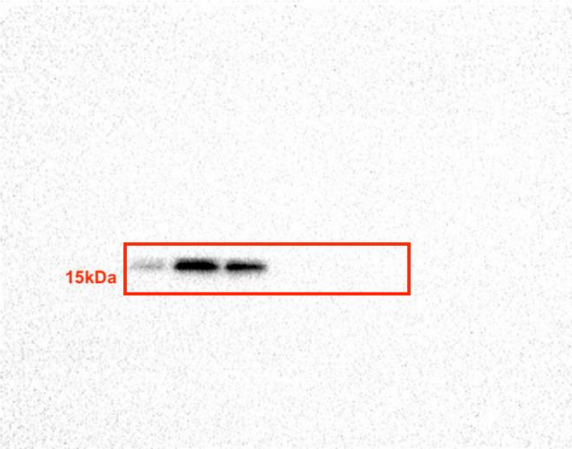

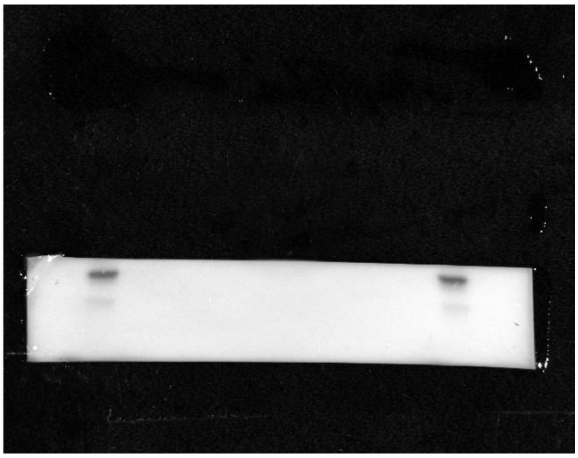

Figure 5F

Anti-β-Tubulin

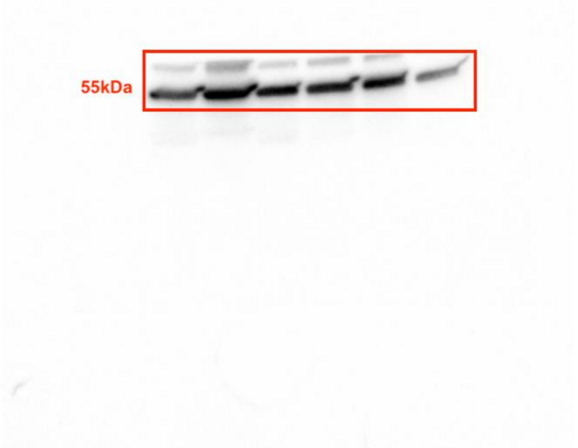

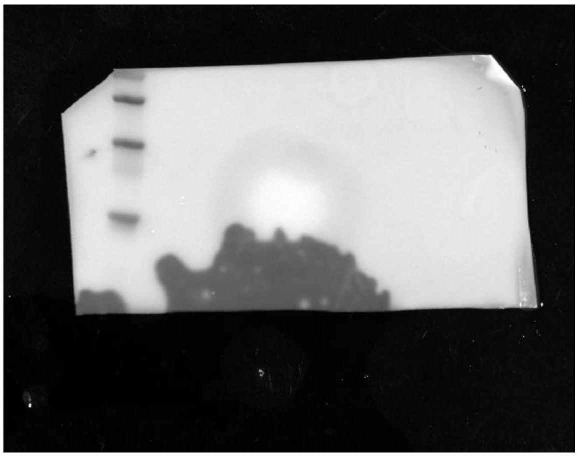

Anti-pBcl2-S70

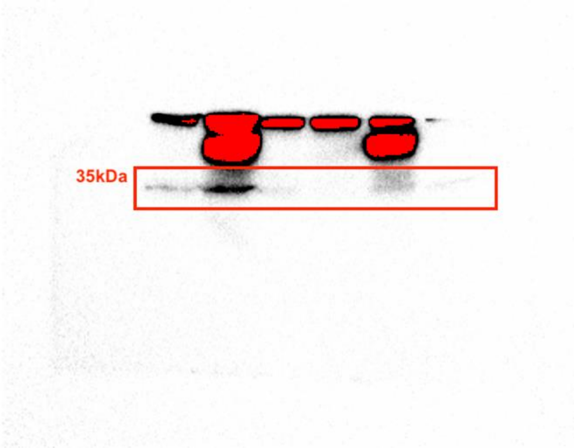

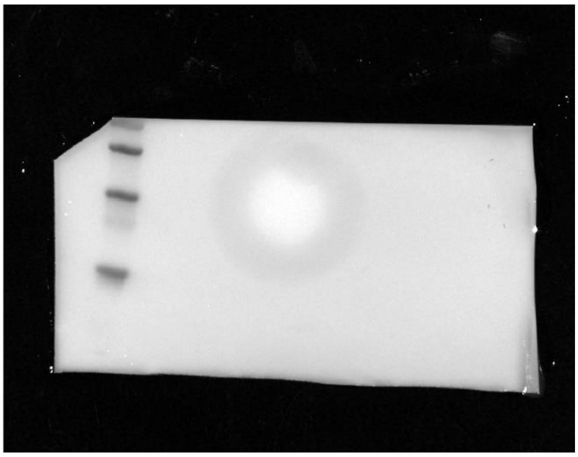

Anti-β-Tubulin

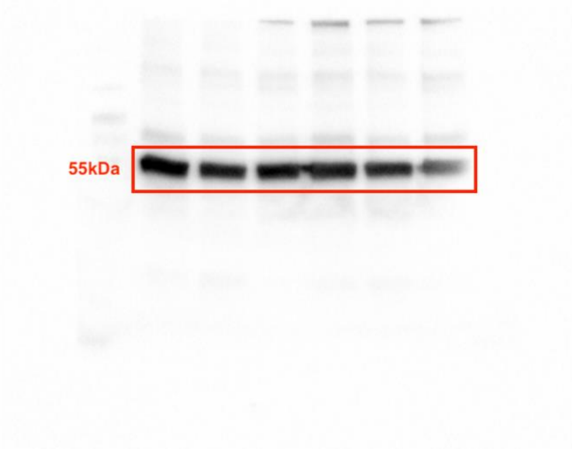

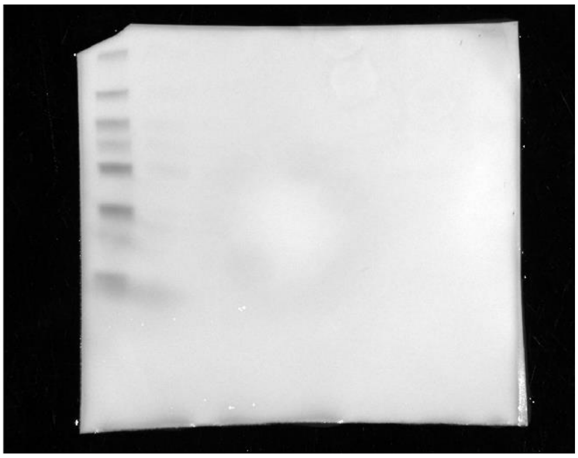

Anti-survivin

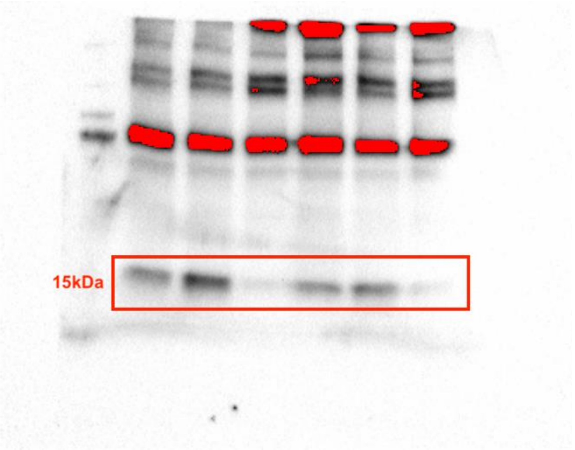

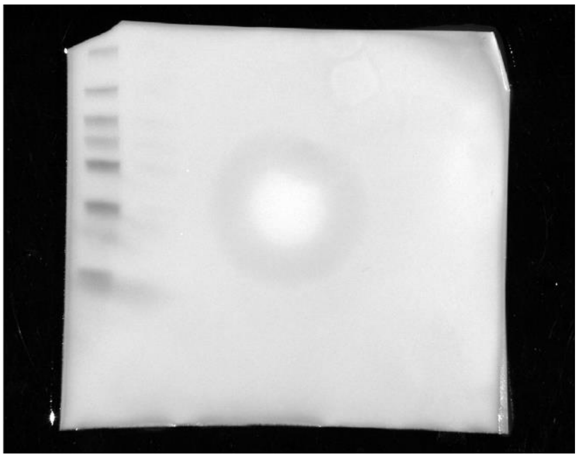

Figure 6I

Anti-COMP

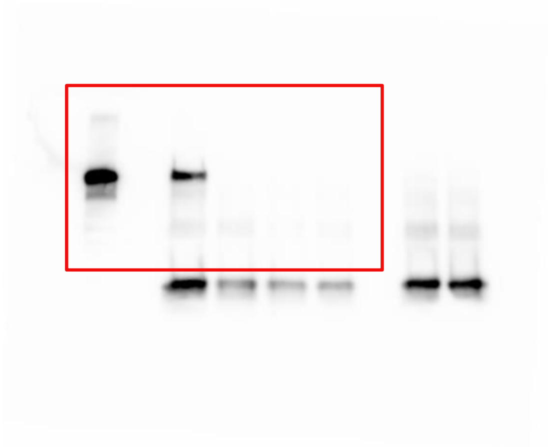

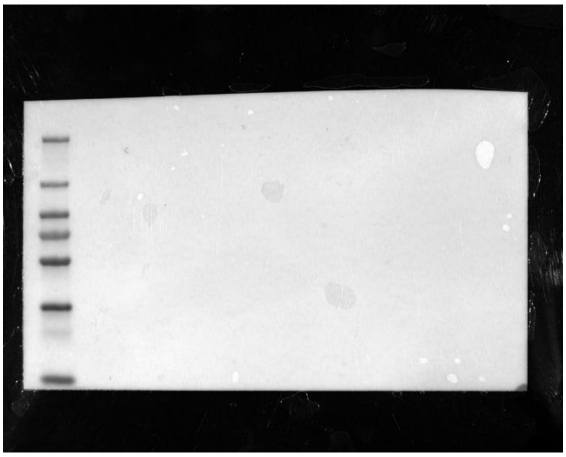

Anti-COMP

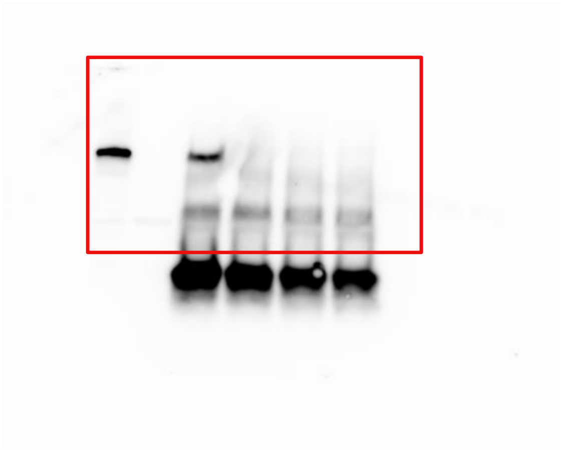

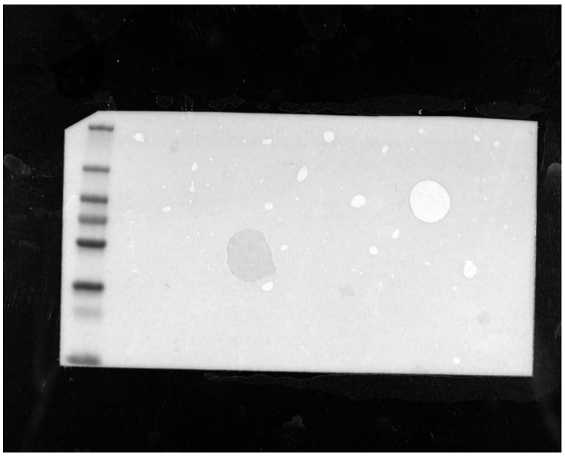

Figure 6K

Anti-calpain

100KDa

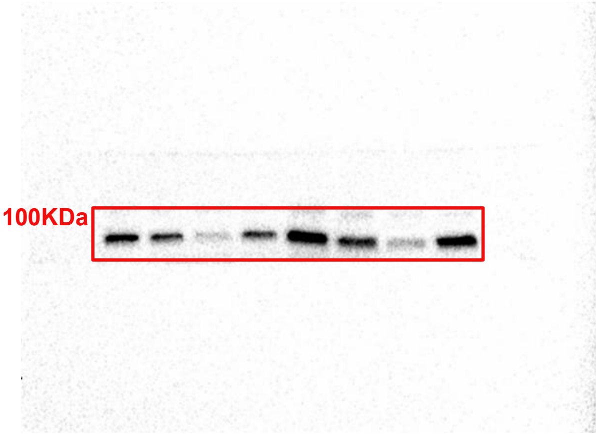

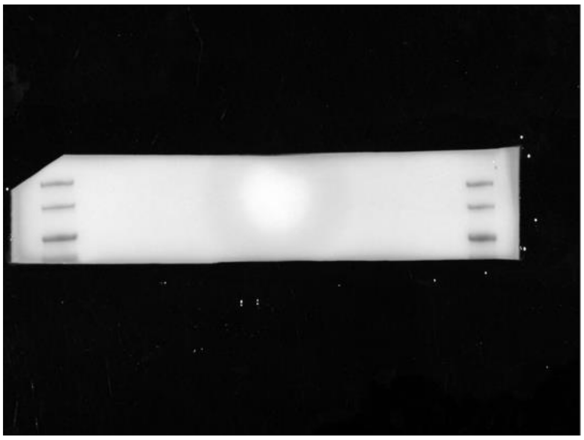

Anti-β-Tubulin

55KDa

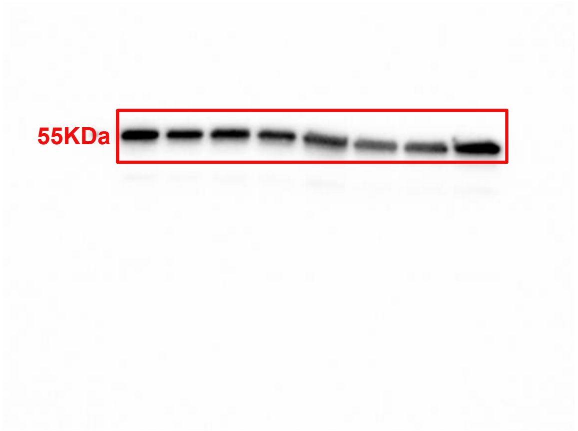

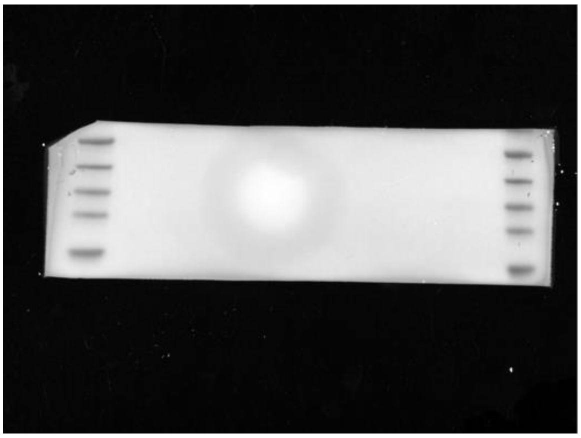

Anti-cleaved caspase 3

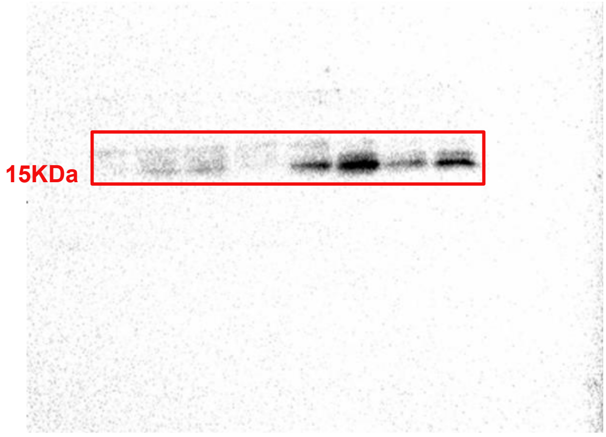

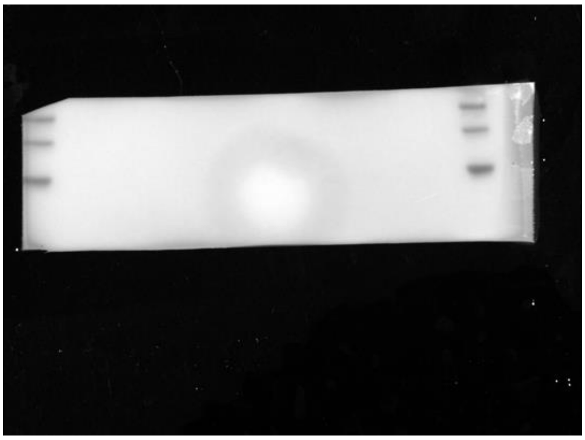

Anti-β-Tubulin

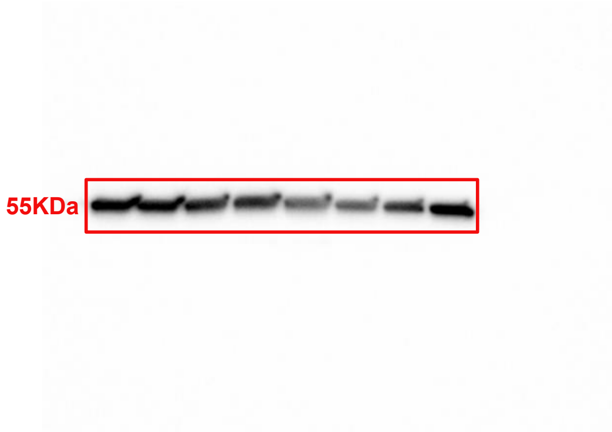

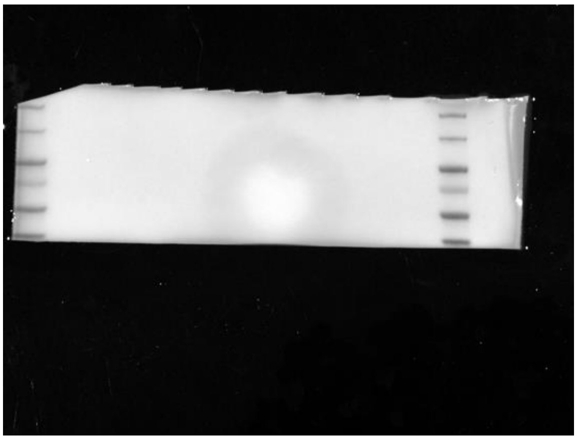

Supplementary Figure S9

Anti-cleaved caspase-8

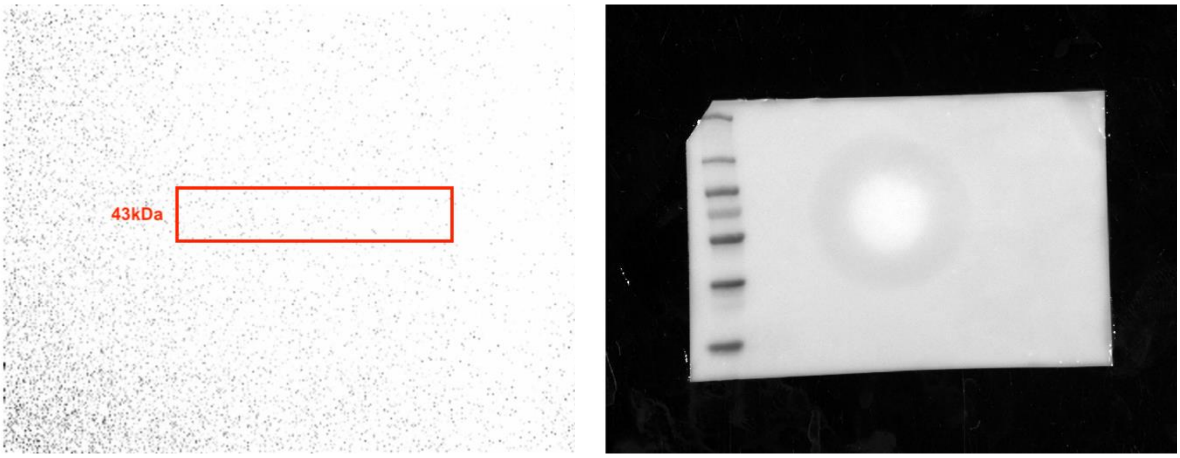

Anti-β-Tubulin

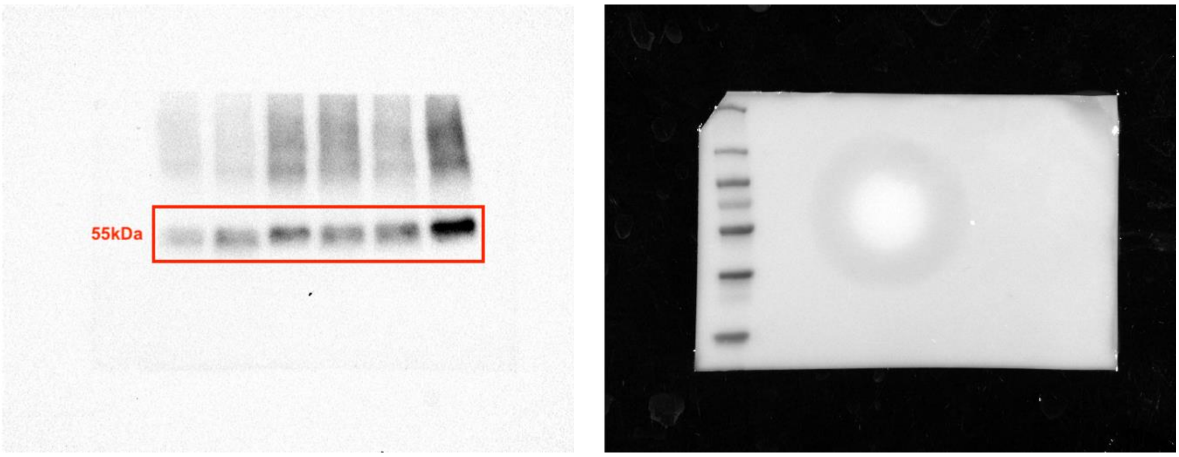

Supplementary Figure S10

Anti-cytochrome C

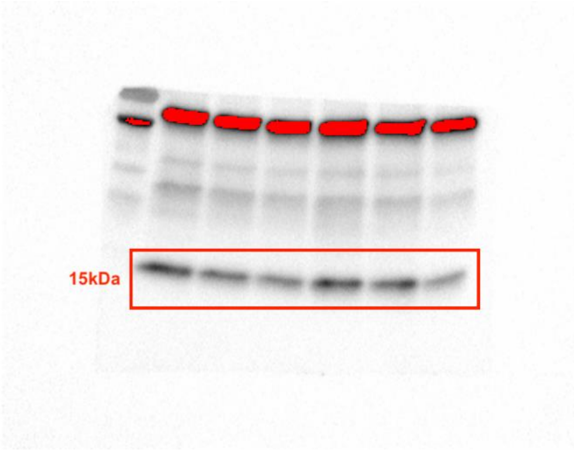

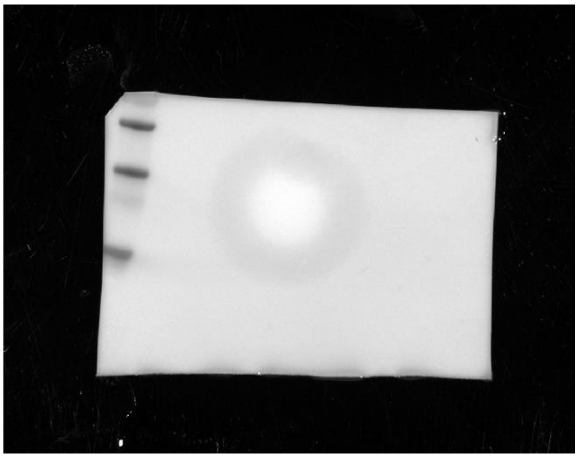

Anti-β-Tubulin

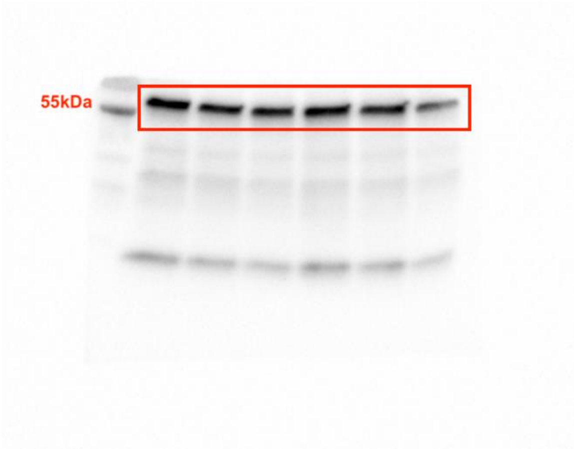

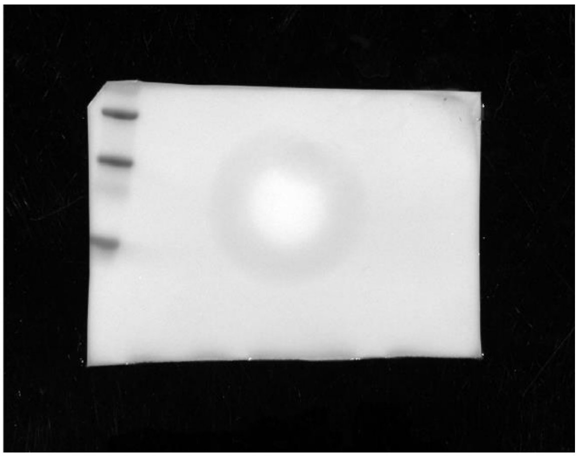

Supplementary Figure S14

Anti-calpastatin

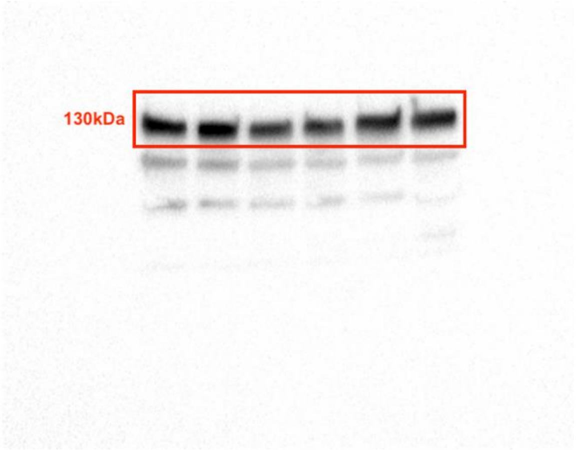

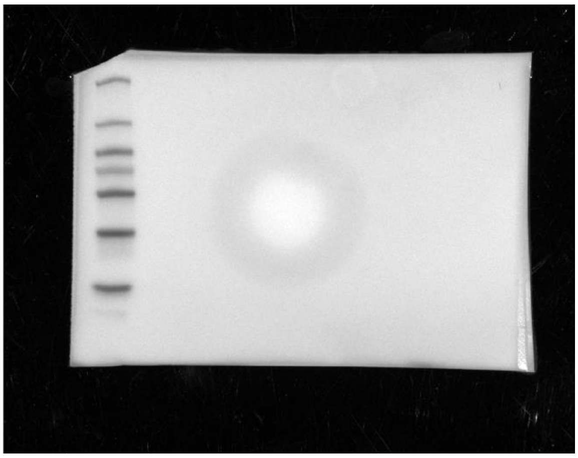

Anti-β-Tubulin

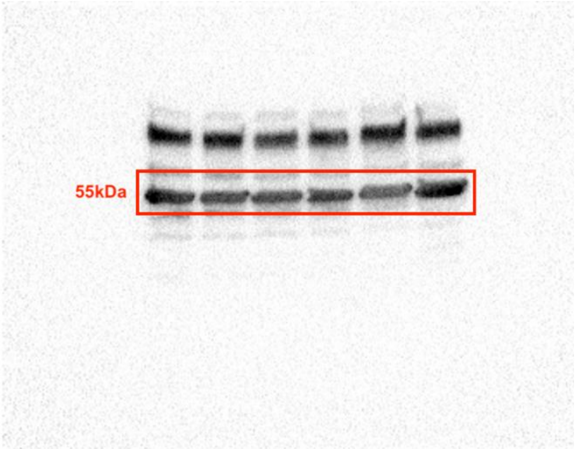

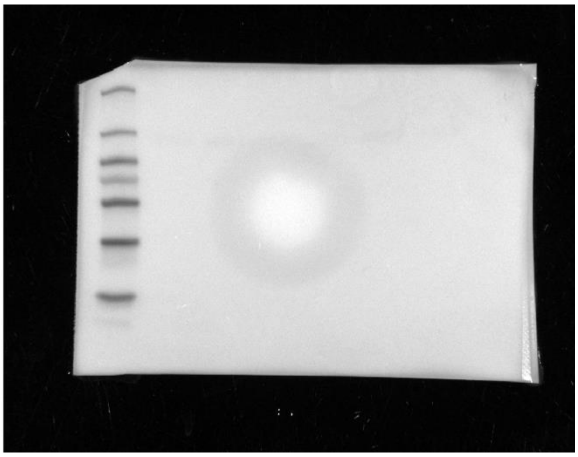

Supplement: Supplementary file 1 — Western blot full length uncropped original version [file 41419_2024_6872_MOESM1_ESM.pdf]
