# Supplementary material for: Intracellular cartilage oligomeric matrix protein augments breast cancer resistance to chemotherapy
Source: Cell Death Dis. 2024 Jul 4;15(7):480. doi: 10.1038/s41419-024-06872-7 (PMC11224260; doi:10.1038/s41419-024-06872-7)
Supplement: Supplementary file 2 — Supplementary data [file 41419_2024_6872_MOESM2_ESM.pdf]

Supporting Information for

Intracellular cartilage oligomeric matrix protein enhances chemoresistance of breast cancer cells.

Hanitrarimalala[1] V., Bednarska[1] I., Murakami[2] T., Papadakos[1, #, *] K. and Blom[1, #] A. M.

[1] Lund University, Department of Translational Medicine, S-214 28 Malmö, Sweden

[2] Saitama Medical University, Department of Microbiology, Saitama 350-0495, Japan

# Contributed equally

Supplementary Tables

Supplementary Table 1. Plasmids constructed and used in this study.			
**Plasmid name**	**Backbone**	**Insert**	**Resistance**
pCDNA3+COMP	pCDNA3	cDNA human COMP	Ampicillin, G418
pCDNA3+COMP-KDEL	pCDNA3	cDNA human COMP (Insertion KDEL)	Ampicillin, G418
pCDNA3+COMPΔ-Cterm	pCDNA3	cDNA human COMP (del. 1552-2271)	Ampicillin, G418
pCDNA3+COMPΔTSP	pCDNA3	cDNA human COMP (del. 802-1551)	Ampicillin, G418
pCDNA3+COMPΔEGF	pCDNA3	cDNA human COMP (del. 220-585)	Ampicillin, G418
pCDNA3+COMPΔ-Nterm	pCDNA3	cDNA human COMP (del. 70-218)	Ampicillin, G418

Supplementary Table 2. Primers used in this study.		
**Name**	**Sequence (5'-3')**	**Description**
COMP+KDEL	GACTATGAGACCCATCAGCTGCGGCAAGCCAAGGACGAGCTGTAGG GACCAGGGTGAGGACCCGCCGGATGAC	Insertion KDEL
MUTMONOF	GCGCGTCCGGACAGGGCCAGGGGATGCAGCAGTCAGTACGCAC	Del. 70-218
MUTDTSPF	CGGCAACGGGATCCTCTGTGGTACCGACTTCAGGGCCTTCCAGAC	Del. 802-1551
COMPstartFWD	AAGCTTCTCCCCGCCACCGCCATGGTCCCC	Start COMP
dC-termREV	GAGAACGCTGAAGTCACGCTCTAGCTCGAG	Del. 1552-2271
dEGF1-4p2FWD	TGTGACGCGTGCGGGCGCGACACTGACCTA	Del. 220-585
dEGF1-4p1REV	CCCGCACGCGTCACACTCCATCACCGTGTT	Del. 220-585
COMPendREV	CTCGAGCTAGGCTTGCCGCAGCAGATGGGT	End COMP

Supplementary Table 3. List of antibodies			
**Target**	**Subtype**	**Supplier**	**Catalogue number**
COMP	Polyclonal Rabbit IgG	Homemade	-
Rabbit IgG HRP	Polyclonal Goat IgG	Dako	P0448
Rabbit IgG HRP	Polyclonal Goat IgG	CST	7074
Cleaved caspase 3	Monoclonal Rabbit	CST	9664
Cleaved caspase 7	Polyclonal Rabbit	CST	9491
Cleaved caspase 8	Monoclonal Rabbit	CST	98134
Cleaved caspase 9	Polyclonal Rabbit	CST	9505
β-Tubulin	Polyclonal Rabbit	Abcam	Ab6046
Calpain	Monoclonal Rabbit IgG	Abcam	Ab108400
Calpain	Monoclonal Mouse	Thermo Fisher Scientific	MA3-940
Mouse IgG1, κ Isotype Ctrl	Monoclonal Mouse	Biolegend	400102
Gamma-H2A.X	Monoclonal Rabbit	Abcam	Ab81299
Survivin	Monoclonal Rabbit	CST	2808
p-Bcl2-Ser70	Monoclonal Rabbit	CST	2827
Calpastatin	Polyclonal Rabbit	Invitrogen	PA587352
GM130	Monoclonal Mouse	BD Transduction	610822
Alexa fluor 488 goat anti-rabbit	Polyclonal Goat IgG	Thermo Fisher Scientific	710 369
Alexa fluor 546 goat anti-rabbit	Polyclonal Goat IgG	Thermo Fisher Scientific	A11035
Alexa fluor 647 goat anti-rabbit	Polyclonal Goat IgG	Thermo Fisher Scientific	A21245
Alexa fluor 546 goat anti-mouse	Polyclonal Goat IgG	Thermo Fisher Scientific	A11003

Supplementary Figures

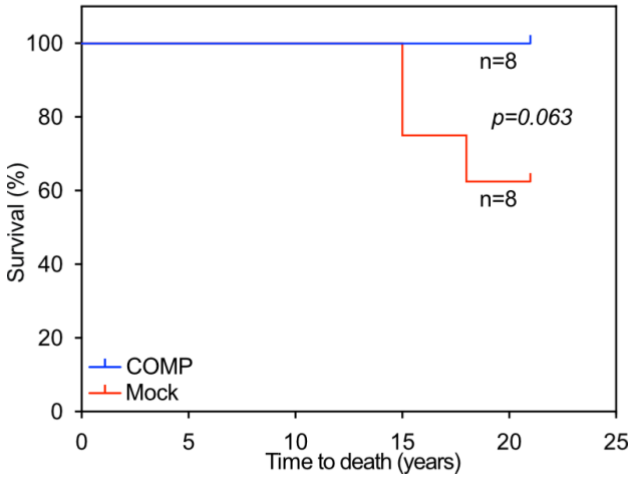

Supplementary Figure S1. Survival curve for the experiment performed in figure 1.

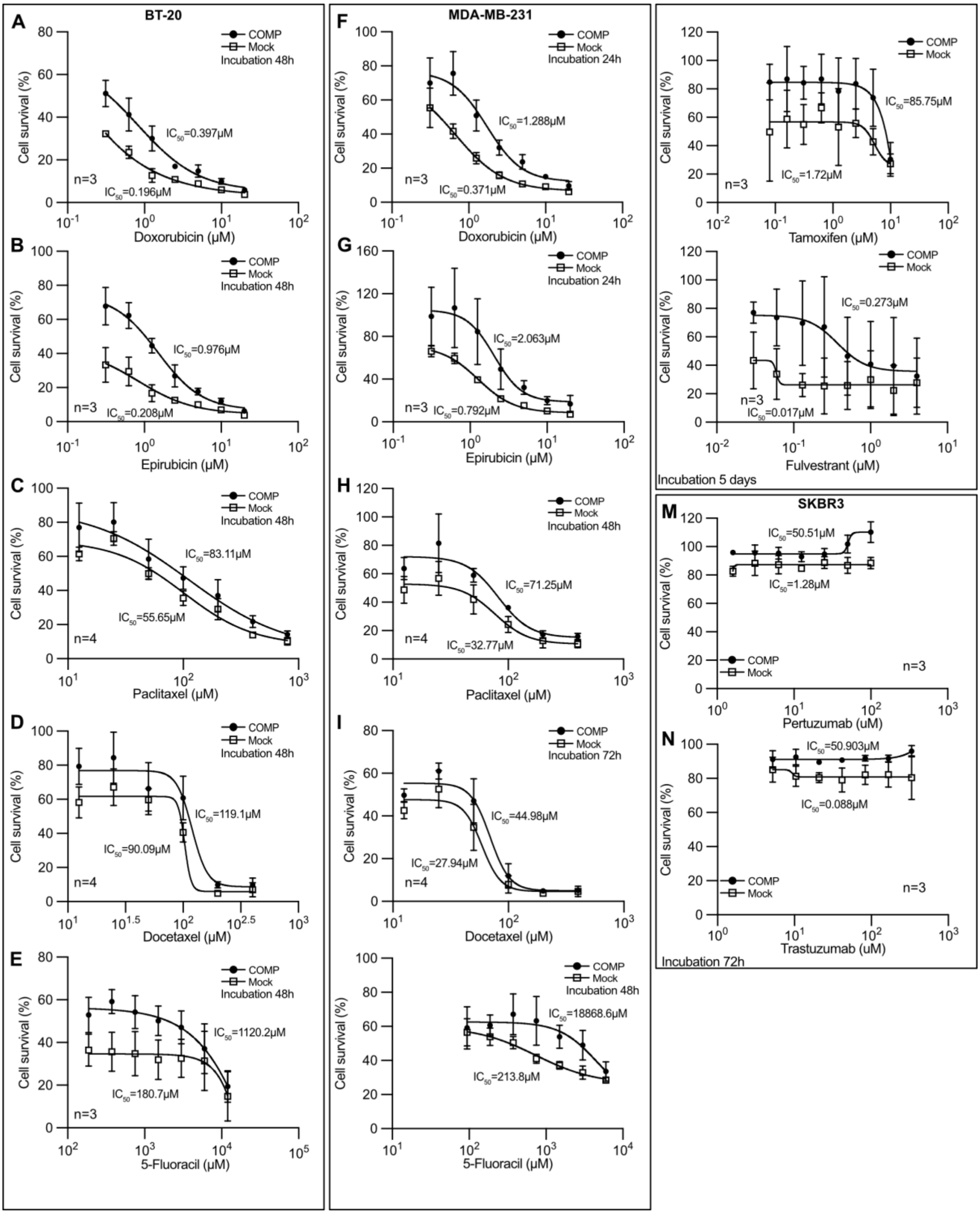

Supplementary Figure S2. Calculation of IC50 from the dose-response curves using data shown in figure 2 **A-N**. The IC50 values for **M** and **N** were calculated to maintain consistency and should be interpreted with caution since the maximum inhibitory dose was not reached.

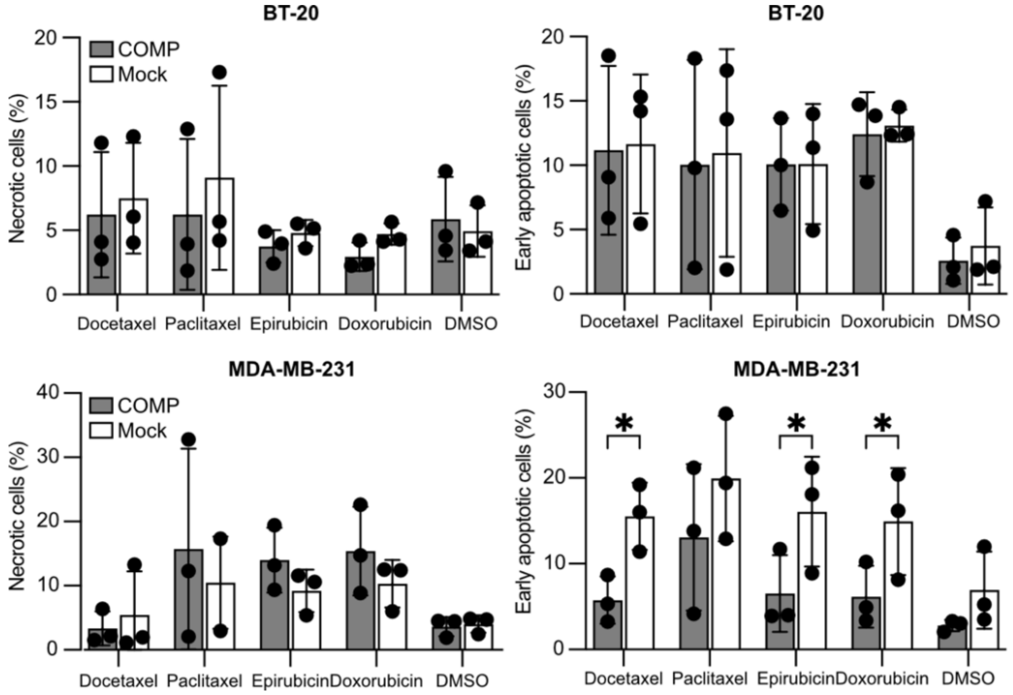

Supplementary Figure S3. Necrotic and early apoptotic cells were quantified by FACS analysis in BT-20 and MDA-MB-231 cells treated with chemotherapy agents using data shown in figure 2 **O-R**.

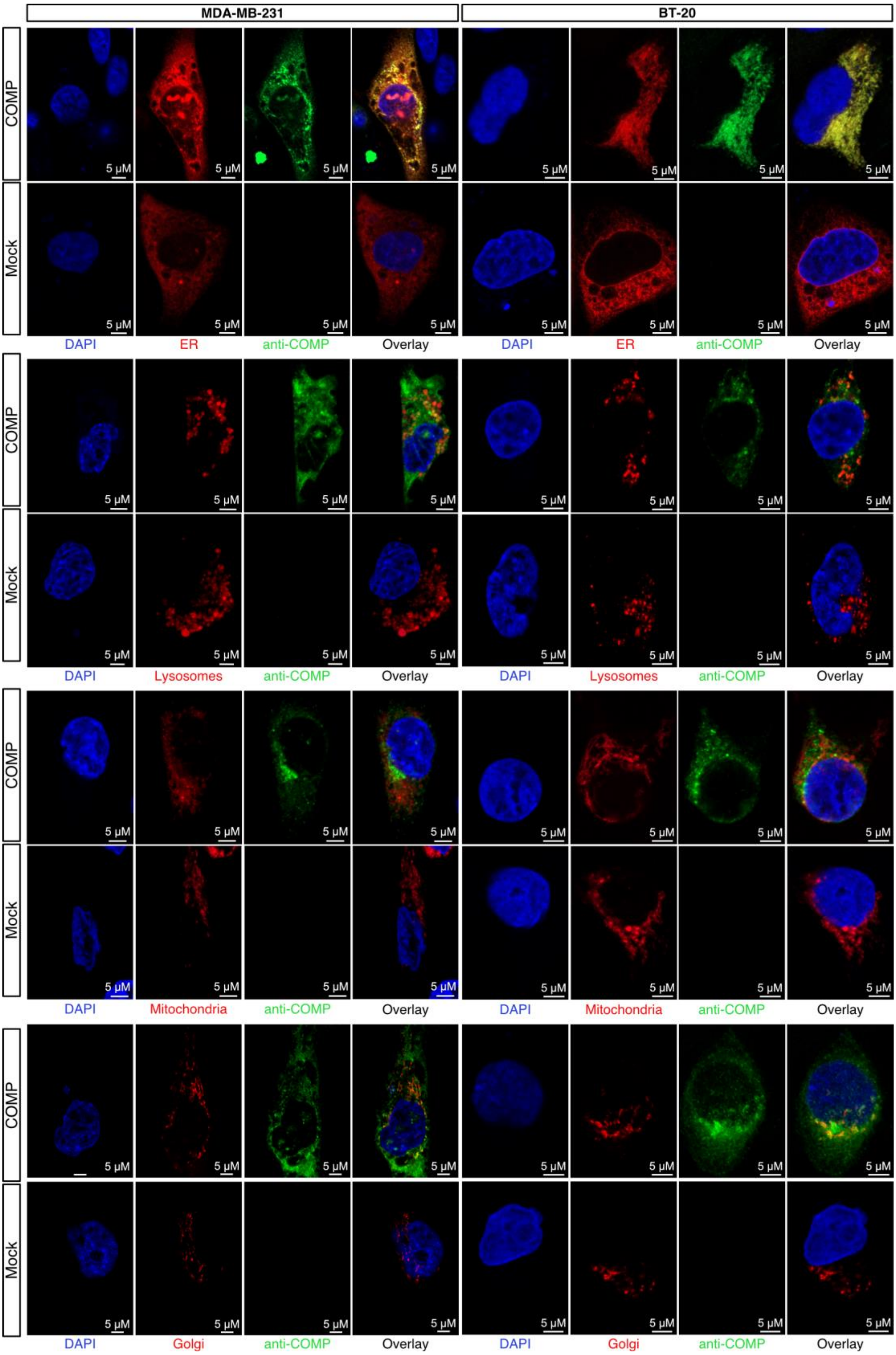

Supplementary Figure S4 Representative confocal microscope images of separated channels of cells stained for the endoplasmic reticulum, lysosomes, mitochondria, Golgi, and COMP. Also refer to figure 3**E.**

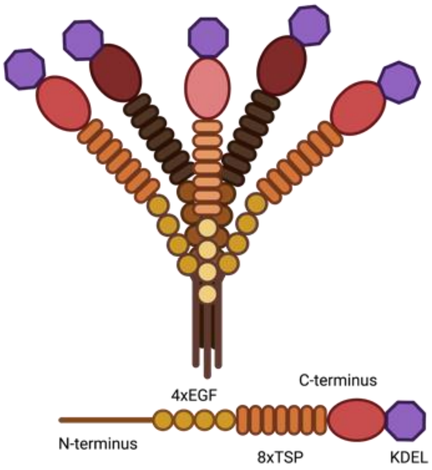

Supplementary Figure S5. Graphic representation of COMP-KDEL protein.

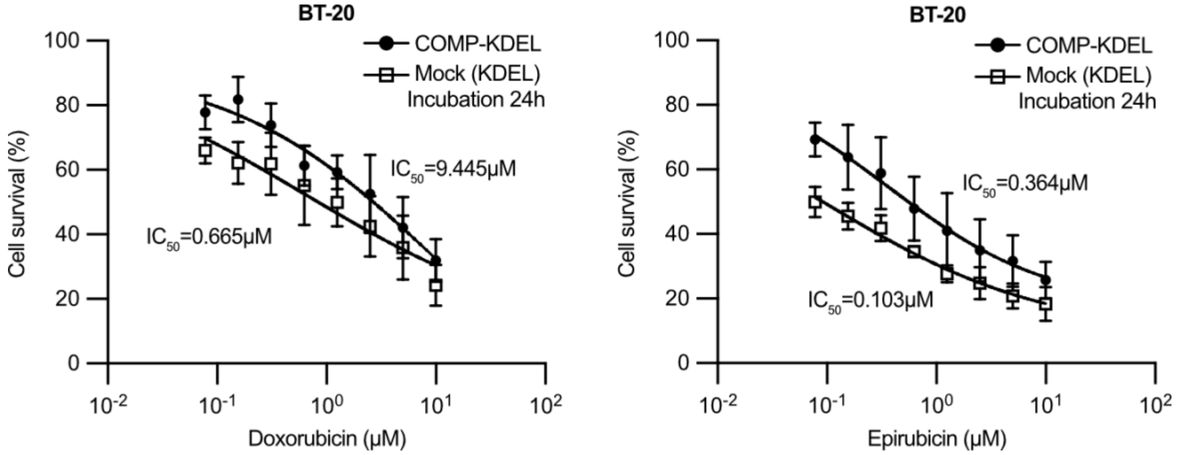

Supplementary Figure S6. Calculation of IC_{50} from the dose-response curves, also refer to figure 3 **H&I**.

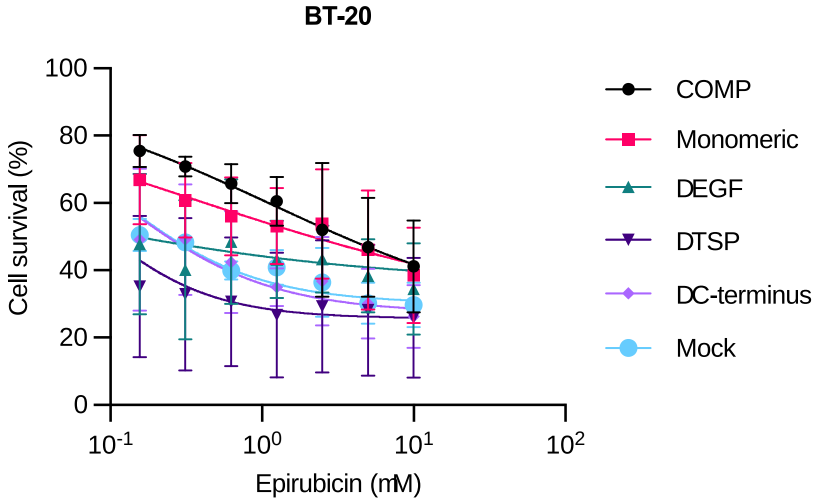

IC_{50} COMP = 1.135µM
IC_{50} Monomeric = 0.272µM
IC_{50} ΔC-terminus = 0.107µM
IC_{50} ΔEGF = 0.011µM
IC_{50} ΔTSP= 0.067µM
IC_{50} Mock = 0.102µM.

Supplementary Figure S7. Percent of inhibition of cells expressing COMP, COMP mutants and mock. also refer to figure 3 **L**.

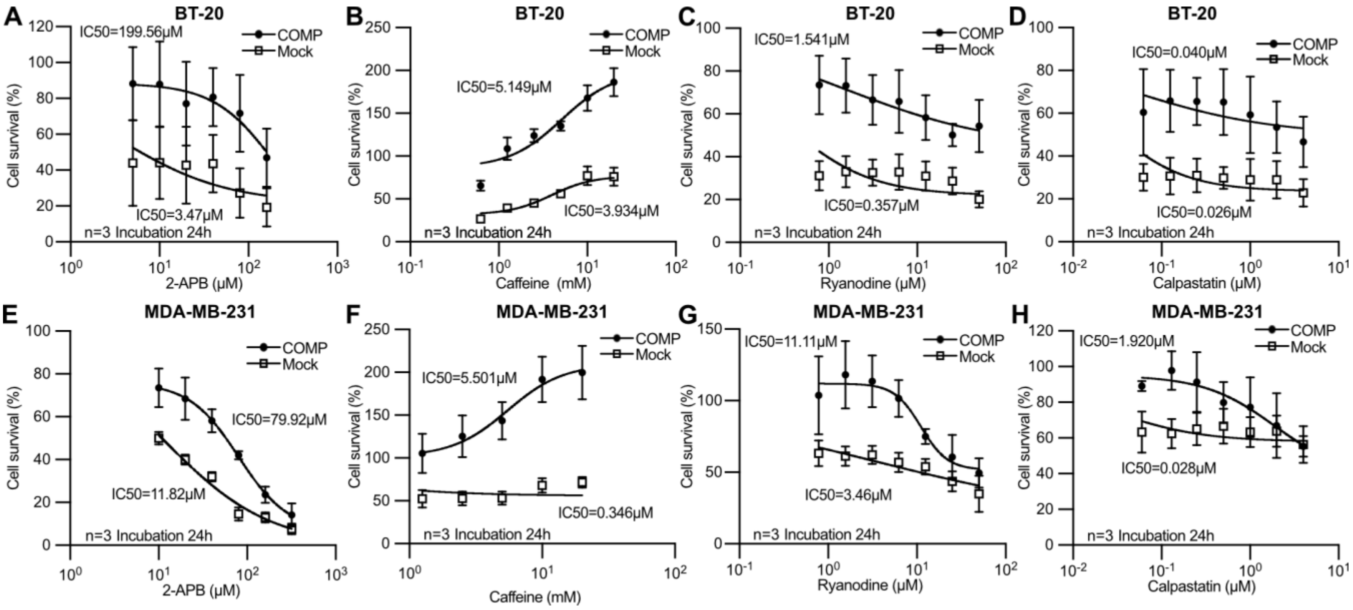

Supplementary Figure S8. Calculation of IC50 from the dose-response curves, also refer to figure 4 **A-H**.

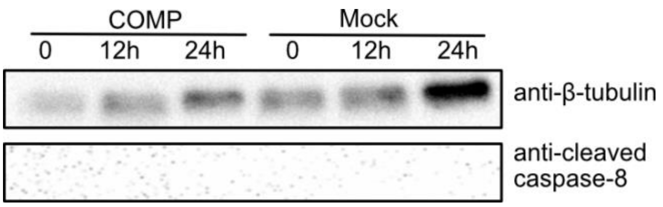

Supplementary Figure S9. Cleaved caspase-8 level via western blot in BT-20 expressing COMP and mock cells following 10 μM epirubicin treatment. n = 3.

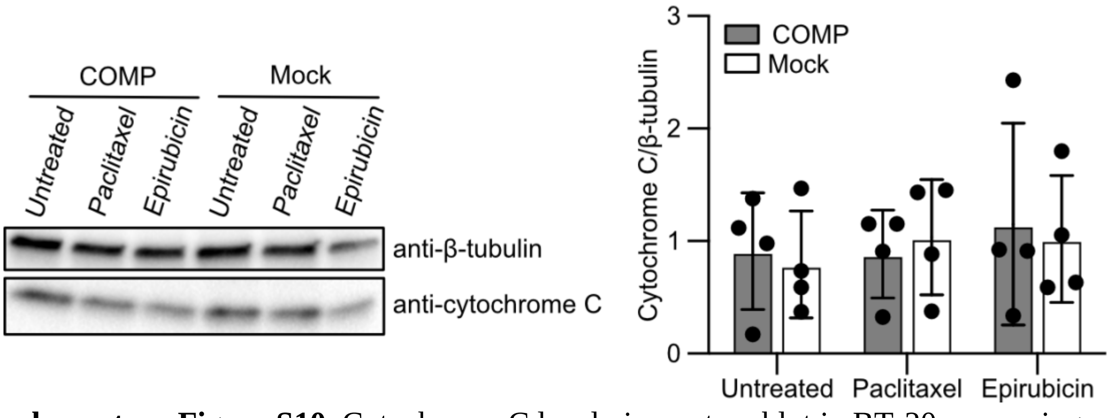

Supplementary Figure S10. Cytochrome C level via western blot in BT-20 expressing COMP and mock cells. n = 4. Statistical significances were determined by two-way ANOVA.

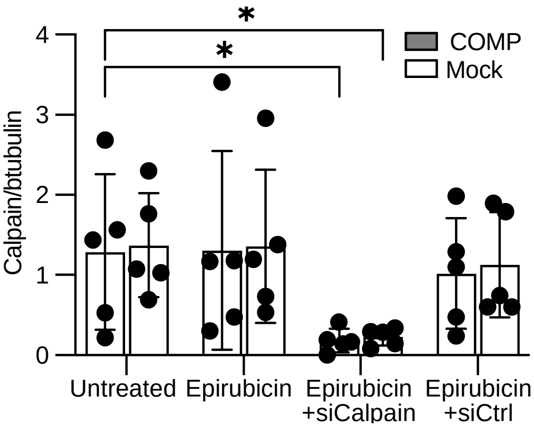

Supplementary Figure S11. Western blot quantification of calpain level following calpain silencing on BT-20 expressing COMP and mock cells. Statistical significances were determined by two-way ANOVA. *p<0.05

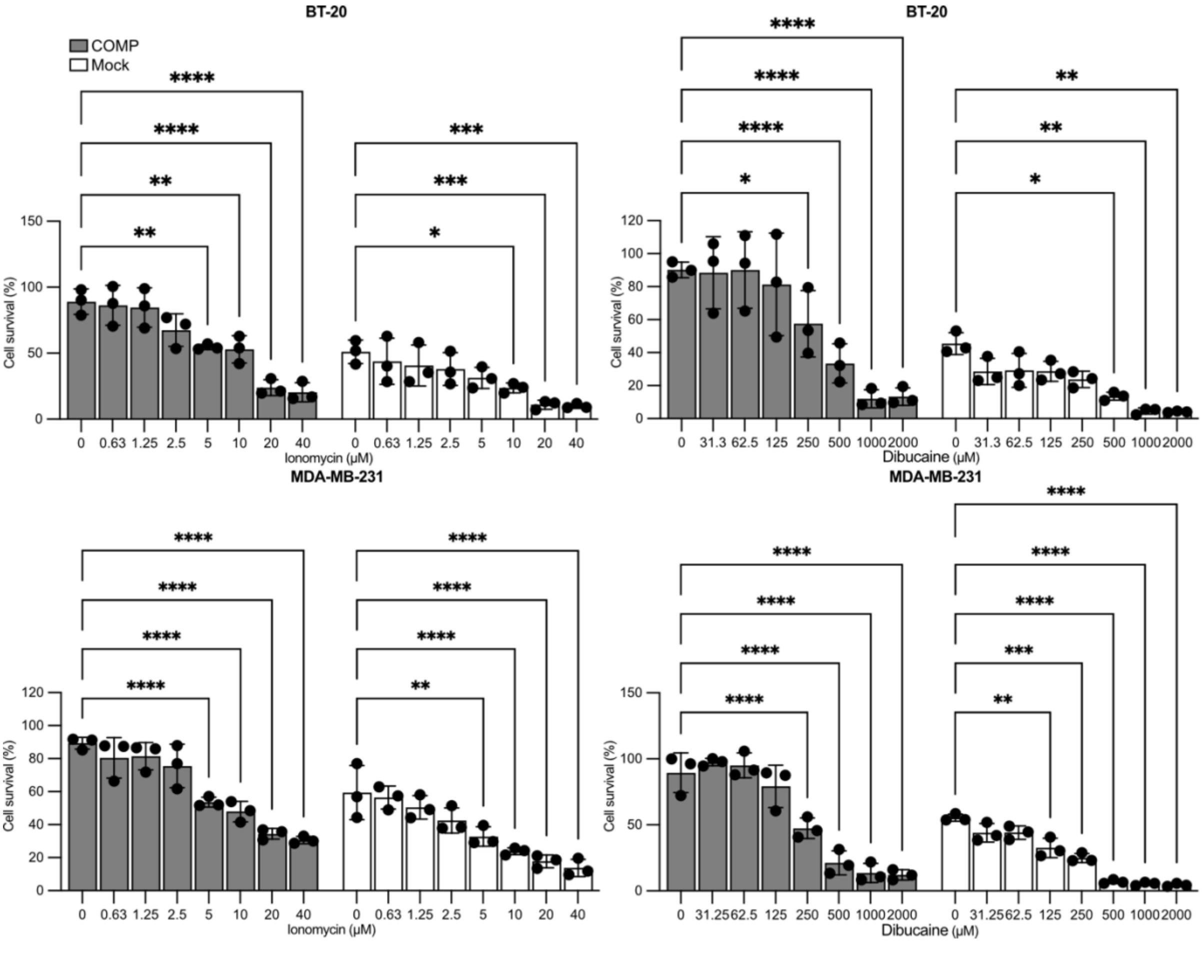

Supplementary Figure S12. Survival assay for Figure 6 **K-N**, in which the statistical comparison is carried out between the untreated COMP-expressing and mock control cells, and those treated with ionomycin or dibucaine.

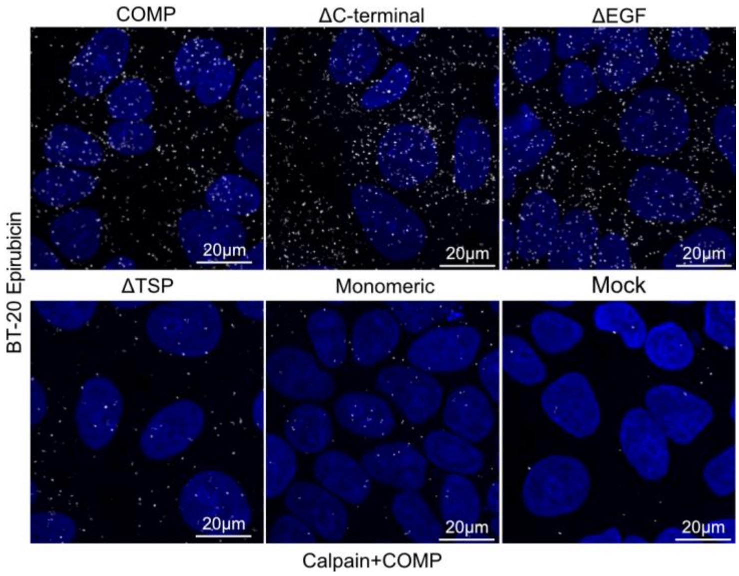

Supplementary Figure S13. Representative images of the PLA assay under epirubicin treatment of figure 6**T.**

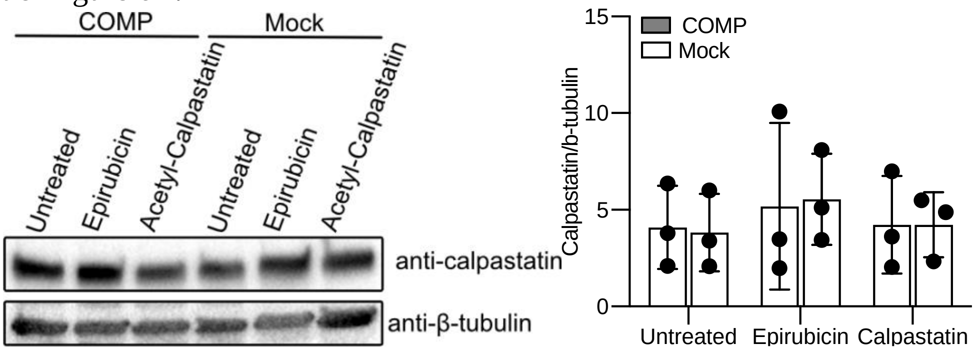

Supplementary Figure S14. Calpastatin level via western blot in BT-20 expressing COMP and mock cells after 24h epirubicin and acetyl-calpastatin treatment. n = 3. Statistical significances were determined by two-way ANOVA.
